# Pharmacological modulation of cGAS-STING-NLRP3 signaling by nano-immunomodulators in Alzheimer and Parkinson disease

**DOI:** 10.1007/s10787-026-02386-0

**Published:** 2026-09-15

**Authors:** Mohammed A. Abdel-Rasol

**Affiliations:** Faculty of Science, Department of Zoology, Ain Shams University, Abbassia, Cairo, 11566 Egypt

**Keywords:** Microglial polarization, NLRP3 inflammasome, cGAS-STING pathway, Polydopamine nanoparticles, Biomimetic nanozymes, Exosome nanocarriers, Nanobody-drug conjugates, Nanotheranostics, Gene-delivering nanoparticles, AI-driven nanomedicine, Brain-on-a-chip, 3D bioprinted brain tissue, Precision nanomedicine

## Abstract

Neuroinflammation is a central driver of neurodegeneration in Alzheimer’s disease (AD) and Parkinson’s disease (PD), driven by interconnected pathways involving microglial state dysregulation, inflammasome activation, cyclic GMP-AMP synthase-stimulator of interferon genes (cGAS–STING) signaling, impaired mitophagy, CD33-TREM2 imbalance, gut-brain axis disruption, and peripheral immune infiltration across a compromised blood–brain barrier (BBB). Conventional anti-inflammatory therapies remain limited by poor CNS delivery, inadequate cell specificity, and the inability to simultaneously modulate multiple inflammatory pathways. Nano-immunomodulators (NIMs) have emerged as a promising strategy to modulate CNS immunity in preclinical models. This review critically evaluates recent advances in NIM-based strategies for AD and PD, including intrinsically therapeutic nanomaterials, targeted delivery systems, and precision gene-regulatory platforms. Intrinsically therapeutic materials, such as polydopamine nanoparticles and biomimetic nanozymes, provide antioxidant and mitochondrial protective functions, whereas BBB-targeted systems, including nanobody-based platforms and engineered exosomes, enhance targeted CNS delivery. Nanotheranostics approaches integrate imaging capabilities with stimuli-responsive release, while precision interceptors aim to simultaneous regulate pathological processes such as amyloid-beta (Aβ)-associated inflammation and cGAS-STING activation. Emerging gene-delivery strategies, including TREM2 mRNA and CD33-targeting lipid nanoparticles, offer opportunities for microglial reprogramming but remain largely restricted to proof-of-concept and early preclinical stages. The review further discusses AI-driven nanomedicine optimization and advanced human-relevant models, including brain-on-a-chip systems and three-dimensional (3D) bioprinting, as tools to improve translational prediction. Currently, AI approaches primarily support candidate selection, formulation optimization, and mechanistic prediction rather than clinical validation. Major challenges for clinical advancement include achieving precise modulation of microglial states beyond the simplified M1/M2 framework, developing context-dependent regulation of NLRP3 and cGAS–STING pathways, establishing gut–CNS therapeutic strategies, improving pluripotent stem cell (iPSC)-based validation platforms, and defining long-term CNS safety frameworks. By integrating molecular mechanisms, AI-assisted nanomedicine, nanoparticle-enabled immune and gene modulation, and advanced preclinical models, this review provides a mechanistic and translational framework for advancing next-generation NIM strategies in AD and PD. This integrated perspective addresses the limitations of previous single-domain analyses focused either on neuroinflammation or nanotechnology and outlines key considerations for translating NIMs from experimental platforms toward clinical applications.

##  Methodology

This narrative review evaluates nano-immunomodulators (NIMs) targeting neuroinflammation in Alzheimer’s disease (AD) and Parkinson’s disease (PD). The initial PubMed search was conducted in January 2026, followed by supplementary targeted searches during manuscript preparation and revision to incorporate relevant literature published later in 2026. The search covered English-language publications from 2020 to 2026.

The search strategy combined disease-related terms, including “neuroinflammation,” “Alzheimer’s disease,” “Parkinson’s disease,” “blood-brain barrier,” “microglial polarization,” “NLRP3 inflammasome,” “cGAS-STING pathway,” “TREM2,” “mitophagy,” and “gut-brain axis,” with technology-related keywords including “nano-immunomodulators,” “polydopamine nanoparticles,” “biomimetic nanozymes,” “exosome nanocarriers,” “nanobody-drug conjugates,” “nanotheranostics,” “gene-delivering nanoparticles,” “AI-driven nanomedicine,” “brain-on-a-chip,” and “3D bioprinted brain tissue.”

Literature selection involved sequential assessment of titles, abstracts, and full-text articles for relevance to the review objectives. Publications were eligible if they addressed (i) neuroinflammatory mechanisms associated with AD or PD, (ii) NIM platforms or enabling technologies relevant to neuroimmune modulation, or (iii) experimental and translational evidence supporting precision nanomedicine approaches. Studies unrelated to AD/PD neuroinflammation, irrelevant to NIM strategies, published in languages other than English, or providing insufficient methodological information were excluded.

Selected studies were organized into four thematic domains: (1) molecular mechanisms of neuroinflammation, (2) NIM platform design and engineering, (3) emerging therapeutic technologies, and (4) translational challenges. The level of evidence was assessed qualitatively according to study type and experimental strength, prioritizing peer-reviewed original studies, mechanistically supported preclinical investigations, and clinically relevant evidence. Conceptual frameworks, computational predictions, and early proof-of-concept technologies were considered separately from experimentally validated approaches.

Because this manuscript is a narrative review rather than a systematic review or meta-analysis, formal risk-of-bias assessment was not performed. However, platform-specific limitations, including synthesis reproducibility, batch variability, protein corona effects, biodegradation, immunogenicity, manufacturing scalability, and long-term CNS safety, were considered during the critical synthesis. Limitations of this review include potential publication and language bias and the predominance of preclinical evidence, as many emerging NIM approaches, including AI-optimized formulations, engineered exosomes, and gene-based microglial reprogramming systems, remain at early translational stages.

## Introduction

Neuroinflammation is a fundamental immune response, primarily mediated by microglia and astrocytes, that becomes chronically pathological in neurodegenerative diseases (NDs) such as Alzheimer's disease (AD) and Parkinson’s disease (PD) (Adamu et al. 2024; Shi and Yong 2025). This pathological shift is characterized by a predominance of pro-inflammatory M1 microglial polarization, reactive astrogliosis, and NLRP3 inflammasome hyperactivation, which collectively drive sustained IL-1β release and accelerate neuronal damage. Furthermore, a compromised blood–brain barrier (BBB) exacerbates CNS degeneration by permitting peripheral immune cell infiltration (Ajoolabady et al. 2025; Xu et al. 2025a; Beltran-Velasco and Clemente-Suárez 2025).

BBB disruption in NDs reflects progressive neurovascular unit dysfunction rather than an isolated structural defect (Sweeney et al. 2018a, 2018b; Uprety et al. 2021). Chronic exposure to Aβ, α-synuclein, mitochondrial injury, and systemic inflammatory mediators activates endothelial cells, pericytes, astrocytes, and microglia, driving oxidative stress, pro-inflammatory cytokine release, and impaired barrier-supportive signaling (Nehra et al. 2022; Lau et al. 2024; Knox et al. 2022). These processes disrupt tight-junction proteins, including claudin-5, occludin, and zonula occludens-1, increase endothelial transcytosis, and weaken the basement membrane through matrix metalloproteinase-mediated extracellular matrix degradation (Ning et al. 2022; Yang et al. 2020; Montagne et al. 2020). Pericyte dysfunction and astrocytic end-foot reactivity further destabilize the endothelium and impair neurovascular coupling, while altered transport and efflux systems reduce BBB selectivity and clearance of neurotoxic molecules (Brown et al. 2019; Díaz-Castro et al. 2023; Storck et al. 2018). Increased BBB permeability then facilitates the entry of plasma proteins, inflammatory mediators, and peripheral leukocytes into the CNS, further activating microglia and sustaining a self-reinforcing cycle of neuroinflammation (Galea 2021; Merlini et al. 2019; Haruwaka et al. 2019).

Because conventional systemic anti-inflammatory therapies often lack sufficient CNS specificity and may produce off-target adverse effects, safer and more precise immunomodulatory strategies are needed (Xu et al. 2025b). Nanomedicine offers a potential strategy to overcome these limitations by using engineered nanomaterials to enhance BBB transport and enable spatially and temporally controlled delivery of immunomodulatory agents to brain-resident glial cells. However, therapeutic efficiency depends on nanoparticle composition, physicochemical properties, and targeting strategies. Nanoplatforms also enable stimulus-responsive and combinatorial delivery of anti-inflammatory agents, neuroprotective compounds, and gene-based therapeutics, supporting their potential use in precision interventions for neurodegenerative diseases (Wang et al. 2025a).

This review provides a mechanistic analysis of nano-immunomodulators (NIMs) as potential therapeutic platforms for AD and PD (Cayero-Otero et al. 2018; Yang 2025; Tziveleka et al. 2025). Although both disorders involve chronic neuroinflammation, their pathological landscapes differ substantially (Heneka et al. 2025; Tansey et al. 2022; De Marchi et al. 2023). AD is characterized primarily by Aβ accumulation and tau pathology, accompanied by neuroinflammatory changes in cortical and hippocampal regions and early BBB disruption (Scheltens et al. 2021; Pascoal et al. 2021; Nation et al. 2019). In contrast, PD is driven mainly by α-synuclein aggregation, nigrostriatal degeneration, and inflammatory remodeling of the substantia nigra and associated basal ganglia circuits (Bloem et al. 2021; Tan et al. 2020; Jewell et al. 2022). These distinct microenvironments shape the timing and nature of immune responses, including peripheral immune-cell infiltration, microglial activation states, and responsiveness to nanomedicine-based therapies (Chen et al. 2023; Paolicelli et al. 2022; Lei et al. 2024a). Accordingly, this review examines shared inflammatory pathways, particularly NLRP3 and cGAS–STING as convergent neuroimmune targets, while addressing their disease-specific roles in AD and PD.

The review is structured around four interconnected objectives. First, it defines the molecular basis of chronic neuroinflammation, including microglial activation states, inflammasome signaling, cGAS–STING activation, impaired mitophagy, gut–brain axis dysregulation, and the CD33–TREM2 regulatory axis, thereby establishing a framework for rational NIM design. Second, it evaluates advances in NIM platforms, including direct immunomodulators, targeted delivery systems, and nanotheranostics, emphasizing their ability to overcome BBB transport limitations, improve cell-type specificity, and enable stimulus-responsive cargo release. Third, it examines emerging technologies that may accelerate translation, including AI-guided nanoparticle optimization, human-relevant preclinical models such as brain-on-a-chip and 3D bioprinting, and gene-delivery strategies for microglial reprogramming. Finally, it identifies major knowledge gaps and translational challenges to define a roadmap for advancing NIM-based precision neuroimmunotherapy toward clinical application. Technologies are considered according to developmental stage, distinguishing clinically evaluated or experimentally validated approaches from preclinical proof-of-concept strategies requiring further optimization and validation.

This review is positioned within the rapidly expanding 2024–2026 literature addressing individual aspects of nanomedicine and neuroinflammation. Recent work has examined nanomaterials as BBB-crossing delivery platforms for Alzheimer’s disease (AD), focusing primarily on drug transport rather than immunomodulatory mechanisms (Kulkarni et al. 2024; Kochman et al. 2026). Similarly, engineered nanoparticles for AD therapy have been reviewed with an emphasis on amyloid-targeted strategies and BBB delivery, while nanotechnology applications in Parkinson’s disease (PD) have been explored across diagnostic and therapeutic contexts (Dong and Zhong 2025; Jia et al. 2025; Yadav et al. 2025). Parallel mechanistic reviews have characterized major inflammatory pathways in neurodegeneration, including NLRP3-driven neuroinflammation (Mustafa et al. 2025) and cGAS–STING signaling in brain aging and neurodegenerative diseases (Zhang et al. 2025a; Li et al. 2025a), but have largely focused on conventional pharmacological modulation (Table 1).

**Table 1:** Comparison of the present review with related publications

Disease coverage	Main biological focus	Nanomedicine focus	BBB / delivery emphasis	AI / advanced models	Translational emphasis	Scope relative to present review	References
CNS disorders, including AD	BBB structure, function, and transport limitations	Nanomaterials for drug delivery	High; nanoparticle-mediated BBB transport	Limited	General CNS-delivery challenges	Focuses on BBB delivery without integrating NIMs with neuroinflammatory pathways	Kulkarni et al.(2024)
AD	Disease-stage-dependent BBB dysfunction	Targeted nanoparticles	High; compares early- and late-stage AD delivery	Limited	Delivery limitations and disease-stage effects	Restricted to BBB-targeted delivery in AD rather than a broader AD/PD neuroimmune framework	Kochman et al.(2026)
AD	AD pathology and therapeutic delivery barriers	Nanomedicine and engineered nanocarriers	High; BBB bypass/crossing strategies	Limited	Delivery barriers and emerging therapies	Does not integrate NIMs with cGAS–STING, NLRP3, mitophagy, and CD33–TREM2 signaling	Dong and Zhong (2025)
AD	Aβ pathology, oxidative stress, neuroinflammation, and neuronal injury	Engineered nanoparticles	Strong	Limited	Preclinical potential and engineering challenges	AD-centered overview without integrated AD/PD comparison across major NIM classes	Jia et al. (2025)
PD	α-Synuclein pathology, diagnosis, and therapy	Diagnostic and therapeutic nanotechnology	Moderate	Limited	Future diagnostic and therapeutic applications	Broad PD nanotechnology review without integrated analysis of shared and disease-specific neuroimmune pathways	Yadav et al. (2025)
Neurodegenerative diseases	NLRP3 inflammasome and its inhibition	Mainly pharmacological inhibitors	Not primary	Limited	NLRP3 as a therapeutic target	Mechanistic NLRP3 review without NIM design, BBB targeting, or nanomedicine-mediated pathway interception	Mustafa et al. (2025)
Neurodegenerative diseases	STING-mediated neuroinflammation	Mainly pharmacological modulation	Not primary	Limited	STING-targeted therapeutic opportunities	Focuses on STING biology rather than integrating cGAS–STING with NIM engineering and multi-pathway delivery	Zhang et al. (2025a)
Brain aging and neurodegenerative diseases	cGAS–STING, mtDNA, aging, and neurodegeneration	Limited nanomedicine emphasis	Not primary	Limited	Mechanistic and therapeutic perspectives	Does not comprehensively address NIM platforms, BBB targeting, AI design, or advanced validation models	Li et al. (2025a)
AD and PD	Microglial state plasticity, NLRP3, cGAS–STING, PINK1/Parkin mitophagy, CD33–TREM2, gut–brain axis, BBB dysfunction, and peripheral immune infiltration	Direct immunomodulators, PDNPs, nanozymes, nanobodies, exosomes, nanotheranostics, pathway interceptors, and gene-delivery LNPs	Integrated analysis of BBB-targeting mechanisms and CNS-selective delivery	Includes AI optimization, brain-on-a-chip, 3D bioprinting, and iPSC models	Includes manufacturing, biodistribution, safety, regulatory barriers, clinical status, and translational readiness	Integrates neuroimmune mechanisms with NIM design and translational development across AD and PD	Present review

*AD* Alzheimer's Disease, *AI* Artificial Intelligence, *Aβ* Amyloid Beta, *BBB* Blood–Brain Barrier, *CD33* Cluster of Differentiation 33, *cGAS* Cyclic GMP-AMP Synthase, *iPSC* Induced Pluripotent Stem Cell, *LNPs* Lipid Nanoparticles, *mtDNA* Mitochondrial DNA, *NIMs* Nano-immunomodulators, *NLRP3* NOD-, LRR- and Pyrin Domain-Containing Protein 3, *PD* Parkinson's Disease, *PDNPs* Polydopamine Nanoparticles, *PINK1* PTEN-Induced Kinase 1, *STING* Stimulator of Interferon Genes, *TREM2* Triggering Receptor Expressed on Myeloid Cells 2

Despite these advances, a critical gap remains in integrating neuroimmune mechanisms with nanomedicine engineering within a unified therapeutic framework. Existing reviews generally address either the molecular mechanisms of neuroinflammation or the design and delivery characteristics of nanoplatforms, with limited analysis of their intersection. This review addresses this gap by mapping major NIM classes including direct immunomodulators, BBB-targeted carriers, nanotheranostics, pathway-specific nanointerceptors, and gene-delivery lipid nanoparticles (LNPs) onto key inflammatory networks such as cGAS–STING–NLRP3, the PINK1/Parkin mitophagy pathway, and the CD33–TREM2 regulatory axis (Table 1). By integrating AI-driven nanoplatform design, advanced preclinical models, molecular mechanisms, and translational considerations, this review provides a comprehensive framework for advancing precision nanomedicine in neurodegenerative diseases.

## Neuroinflammation mechanisms and pathways

### Microglial state plasticity

Microglial activation in AD and PD is increasingly recognized as a dynamic, heterogeneous process rather than a binary transition between M1 and M2 phenotypes (Ransohoff 2016; Deczkowska et al. 2020; Gao et al. 2025a; Keren-Shaul et al. 2017). Although the M1/M2 classification provides a simplified distinction between pro-inflammatory and reparative responses, single-cell and transcriptomic analyses have identified a spectrum of context-dependent microglial states (Mathys et al. 2019; Hammond et al. 2019; Krasemann et al. 2017). These include homeostatic microglia, disease-associated microglia (DAM), activated response microglia (ARM), interferon-responsive populations, and other transcriptionally distinct phenotypes shaped by disease progression and local environmental signals (Yang et al. 2025a; Friedman et al. 2018; Xue and Wu 2025; Varsamos et al. 2025).

Pro-inflammatory stimuli, including IFN-γ and TNF-α, induce microglial programs involving NF-κB and STAT1 signaling, cytokine production, reactive oxygen species generation, and inflammasome activation. In contrast, PPAR-γ, TGF-β, and TREM2 signaling supports phagocytosis, metabolic adaptation, homeostatic functions, and tissue repair (Varsamos et al. 2025; Shi et al. 2026; Guo et al. 2024a; Masuda et al. 2020). These states are neither discrete nor mutually exclusive, as individual microglia may simultaneously exhibit inflammatory, phagocytic, metabolic, and repair-associated transcriptional programs depending on disease stage and microenvironmental conditions (Li and Barres 2018; Rana et al. 2026).

**Fig. 1:**
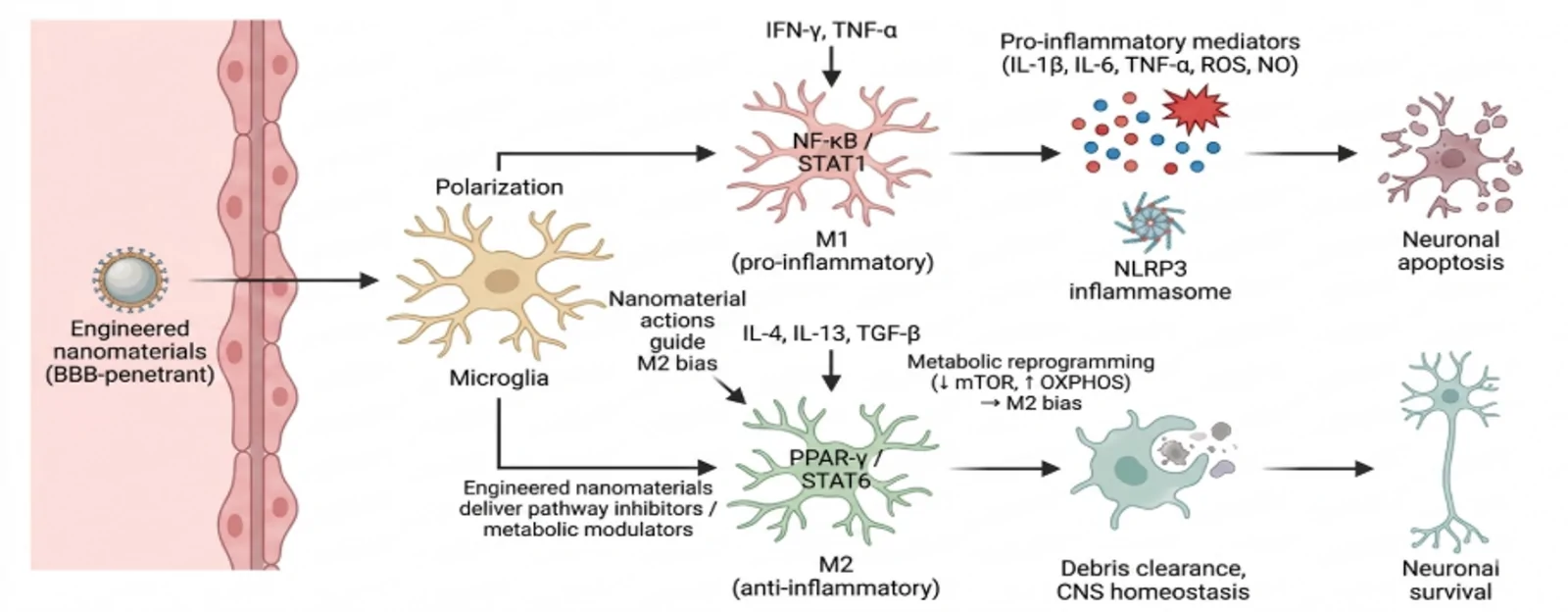
Microglial Polarization in Alzheimer's and Parkinson's Disease: M1/M2 Balance and Therapeutic Strategies. This schematic illustrates the simplified M1/M2 framework of microglial activation in AD and PD. Pro-inflammatory M1-associated microglia, induced by IFN-γ and TNF-α through NF-κB/STAT1 signaling, produce inflammatory mediators including IL-1β, IL-6, TNF-α, ROS, and NO, promote NLRP3 inflammasome activation, and contribute to neuronal injury. In contrast, M2-associated microglial programs, stimulated by IL-4, IL-13, and TGF-β through PPAR-γ/STAT6 and mTOR-associated pathways, support phagocytosis, Aβ clearance, and tissue homeostasis. The figure further depicts how nano-immunomodulators may enable targeted delivery of pathway inhibitors or metabolic modulators across the BBB to regulate inflammatory microglial responses and promote neuroprotective states. Abbreviations: IFN-γ: Interferon-gamma, IL-1β: Interleukin-1 beta, mTOR: Mechanistic Target of Rapamycin, NF-κB: Nuclear Factor kappa-light-chain-enhancer of activated B cells, NLRP3: NOD-like receptor family pyrin domain containing 3, NO: Nitric Oxide, PPAR-γ: Peroxisome Proliferator-Activated Receptor Gamma, ROS: Reactive Oxygen Species, STAT1: Signal Transducer and Activator of Transcription 1, TGF-β: Transforming Growth Factor-beta, TNF-α: Tumor Necrosis Factor-alpha

Accordingly, therapeutic strategies should move beyond the simplified goal of inducing an M1-to-M2 transition and instead regulate microglial state balance. Engineered nanomaterials may help reshape microglial functional landscapes by suppressing persistent inflammatory signaling while supporting homeostatic, phagocytic, and repair-associated programs across the microglial continuum (Yadav et al. 2025; Heneka et al. 2018) (Fig. 1).

### Inflammasome activation

The NLRP3 inflammasome, comprising NLRP3, the adaptor protein ASC, and caspase−1, is a central mediator of chronic neuroinflammation in neurodegenerative diseases (Mustafa et al. 2025; Voet et al. 2019; Halle et al. 2008). Disease-associated protein aggregates can trigger its activation, with Aβ-driven signaling particularly implicated in AD and α-synuclein-associated activation more prominent in PD (Ising et al. 2019; Codolo et al. 2013; Gan et al. 2026). Mitochondrial dysfunction and ROS further enhance inflammasome activity by promoting mitochondrial damage and mtDNA release (Moustakli et al. 2025; Zhou et al. 2011; Shi et al. 2015).

Following inflammasome assembly, caspase−1 processes pro-IL-1β and pro-IL-18 into their mature inflammatory forms and cleaves gasdermin D (GSDMD), initiating pyroptosis (Xu et al. 2025b; Kayagaki et al. 2015; Ding et al. 2016). The resulting GSDMD N-terminal fragment (GSDMD-NT) forms membrane pores that disrupt cellular integrity, causing pyroptotic cell death and release of IL-1β, IL-18, and damage-associated molecular patterns (DAMPs), including HMGB1, extracellular ATP, and mtDNA (Kaczmarek et al. 2013; Bours et al. 2006). These signals activate pattern-recognition and purinergic receptors on neighboring microglia and astrocytes, further promoting inflammasome priming and activation (Liddelow and Barres 2017; Broz and Dixit 2016).

Non-canonical inflammasome signaling provides an additional route to GSDMD-mediated pyroptosis (Kesavardhana et al. 2020). Murine caspase−11 and its human counterparts, caspase−4 and caspase−5, directly cleave GSDMD independently of caspase−1 and can subsequently promote NLRP3 activation (Franklin et al. 2014). Together, these pathways establish a self-amplifying inflammatory circuit in which pyroptotic cell death drives cytokine and DAMP release, sustaining inflammasome activation in surrounding cells (Decout et al. 2021).

**Fig. 2:**
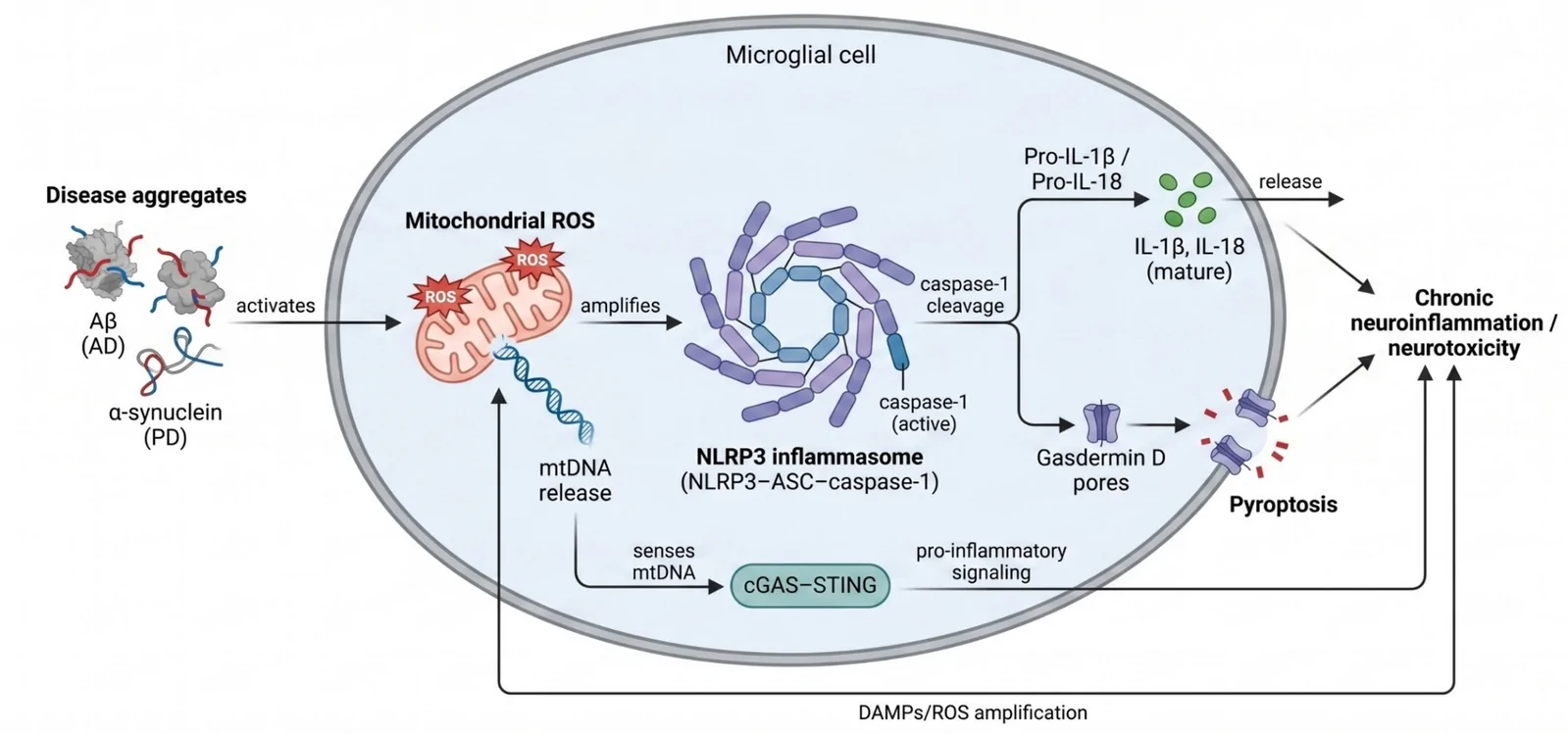
NLRP3 Inflammasome Activation Pathway in Alzheimer’s and Parkinson’s Diseases. This figure illustrates the activation of the NLRP3 inflammasome in microglia by disease-associated aggregates, including Aβ in AD and α-synuclein in PD, together with ROS-driven mitochondrial dysfunction. NLRP3 assembly with the adaptor protein ASC promotes caspase−1 activation, resulting in the maturation of IL-1β and IL-18 and induction of GSDMD-mediated pyroptosis. Subsequent release of inflammatory cytokines and DAMPs further amplifies neuroinflammation and activates downstream cGAS–STING signaling, establishing a self-reinforcing inflammatory circuit. The schematic also highlights potential NIM intervention strategies, including direct NLRP3 inhibition and ROS scavenging, to attenuate inflammasome-driven neuroinflammatory responses. Abbreviations: Aβ: Amyloid beta, AD: Alzheimer's Disease, ASC: Apoptosis-associated speck-like protein containing a CARD, cGAS–STING: Cyclic GMP-AMP Synthase Stimulator of Interferon Genes, DAMPs: Damage-Associated Molecular Patterns, GSDMD: gasdermin D, IL-1β: Interleukin-1 beta, IL-18: Interleukin-18, mtDNA: Mitochondrial DNA, NLRP3: NOD-, LRR- and pyrin domain-containing protein 3, PD: Parkinson's Disease, ROS: Reactive Oxygen Species

Persistent NLRP3 activation contributes to chronic neuroinflammation and intersects with mtDNA-dependent cGAS–STING signaling, linking inflammasome activity to broader innate immune dysregulation and neurotoxicity (Pfaffinger et al. 2025) (Fig. 2).

### Gut-brain axis

Dysbiosis of the gut-brain axis systemically drives neurodegeneration (Wang et al. 2025a; Chen et al. 2025). Environmental or dietary stressors enrich pathogenic bacteria that secrete lipopolysaccharides (LPS) and deplete neuroprotective short-chain fatty acids (SCFAs) (Palanivelu et al. 2025; Liu et al. 2025a). This metabolic imbalance elevates systemic cytokines (TNF-α, IL-6, IL-1β), degrades endothelial tight junctions, and compromises the BBB, facilitating the CNS influx of peripheral immune cells (Loh et al. 2024; Chakrabarti and Chattopadhyay 2025).

**Fig. 3:**
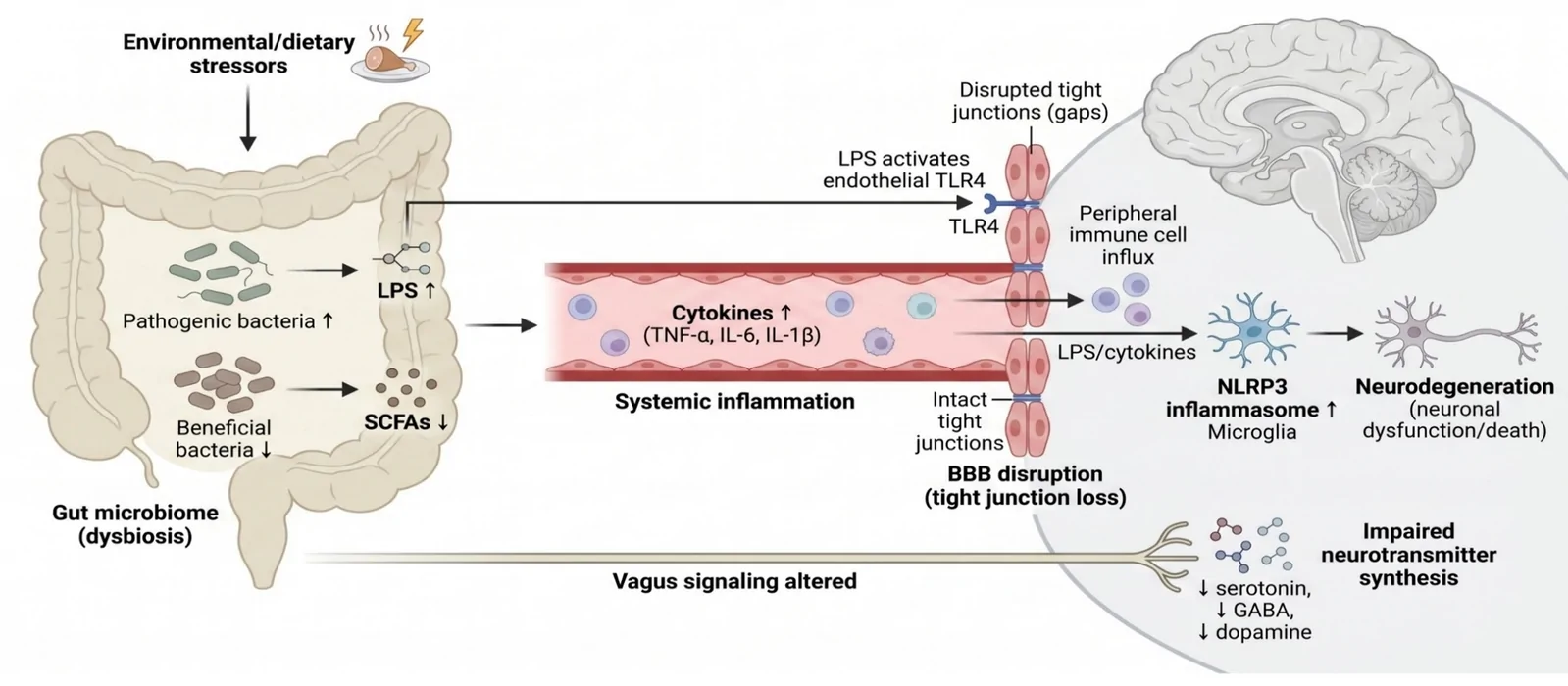
Gut-Brain Axis: Dysbiosis-Driven Cascade to Neurodegeneration. This schematic illustrates how gut microbiota dysbiosis contributes to CNS pathology through interconnected immune, vascular, and neural mechanisms. Expansion of pathogenic bacterial populations and depletion of SCFA-producing species increase systemic LPS levels and pro-inflammatory cytokines, including TNF-α, IL-6, and IL-1β, leading to endothelial tight-junction disruption and BBB impairment. These alterations facilitate peripheral immune-cell infiltration, while disrupted vagal signaling affects neurotransmitter regulation, including serotonin, GABA, and dopamine pathways. Gut-derived LPS further activates endothelial TLR4 signaling and microglial NLRP3 inflammasomes, sustaining neuroinflammation and promoting neurodegenerative processes. The figure highlights the gut–brain–immune axis as a potential multi-level target for future nano-immunomodulatory interventions. Abbreviations: BBB: Blood–Brain Barrier, GABA: Gamma-Aminobutyric Acid, IL-1β: Interleukin-1 beta, IL-6: Interleukin-6, LPS: Lipopolysaccharides, NLRP3: NOD-, LRR- and pyrin domain-containing protein 3, SCFAs: Short-Chain Fatty Acids, TNF-α: Tumor Necrosis Factor-alpha, TLR4: Toll-like Receptor 4

Furthermore, altered vagal signaling impairs neurotransmitter synthesis (serotonin, GABA, dopamine) (Santos et al. 2023; Yang et al. 2023). Gut-derived LPS also directly activates endothelial Toll-like receptors and microglial NLRP3 inflammasomes, establishing the microbiome as a critical therapeutic target (Cai et al. 2026; Zhang et al. 2025b) (Fig. 3).

### Peripheral immune cell infiltration

BBB disruption facilitates pathological recruitment of peripheral leukocytes into the CNS (Tumpa et al. 2025; Huang et al. 2021). This breakdown reflects coordinated neurovascular unit dysfunction, in which inflammatory cytokines and oxidative stress impair endothelial tight-junction proteins, including claudin-5, occludin, and zonula occludens-1 (Langen et al. 2019). Concurrent matrix metalloproteinase activity degrades the basement membrane and extracellular matrix, while pericyte dysfunction, astrocytic end-foot impairment, increased transcellular transport, and altered efflux mechanisms further reduce BBB selectivity (Yuan et al. 2025). Collectively, these changes increase the entry of circulating immune cells and inflammatory mediators, thereby amplifying CNS inflammation (Santos-Lima et al. 2022).

Among infiltrating immune populations, neutrophils are recruited early through CXCL1 and IL-8 signaling and contribute to oxidative stress and the release of neutrophil extracellular traps (NETs) (Nowaczewska-Kuchta et al. 2025; Liu et al. 2025b). T cells subsequently infiltrate the parenchyma, producing granzyme B and promoting pro-inflammatory microglial polarization (Yang et al. 2025b; Van Hove et al. 2025). In parallel, CCL2/CX3CL1 signaling facilitates monocyte recruitment and differentiation into macrophage-like cells that further sustain cytokine production (Tian et al. 2025a; Nie et al. 2025; Dong et al. 2025). Nanomedicine-based approaches may modulate these processes by targeting chemokine pathways, such as CXCR1/2 and CCR2, or by enabling targeted delivery of immune-regulatory agents, including PD-1 and CTLA-4 modulators, to reduce infiltration-associated neuroinflammation (Jia et al. 2025; Shokr 2025; Ma et al. 2025a) (Table 2).

**Table 2:** Characteristics of peripheral immune cell infiltration and nanomedicine interventions in the CNS

Entity	Recruitment signals / targets	Timing & localization	Pathological role / mechanism of action	References
Neutrophils	CXCL1, IL-8	Early recruitment	Induce oxidative stress; release cytotoxic NETs	Nowaczewska-Kuchta et al.( 2025), Liu et al. (2025b)
T-cells	Not specified	Subsequent migration into the parenchyma	Secrete granzyme B; reinforce microglial M1 polarization	(Yang et al. (2025b), Van Hove et al. (2025)
Monocytes	CCL2/CX3CL1 gradients	Simultaneous attraction (with T-cells)	Differentiate into cytokine-amplifying macrophage-like cells	Tian et al. (2025a), Nie et al. (2025), Dong et al. (2025)

*BBB* Blood–brain barrier, *CCL2* C–C motif chemokine ligand 2, *CCR2* C–C chemokine receptor type 2, *CNS* Central nervous system, *CTLA-4* Cytotoxic T-lymphocyte-associated protein 4, *IL-8* Interleukin-8, *NETs* Neutrophil extracellular traps, *PD-1* Programmed cell death protein 1

### Metabolic reprogramming and mitochondrial quality control

Microglial metabolism and mitochondrial quality control strictly dictate neurotoxicity (Huang et al. 2024; Nirakis et al. 2025). Pro-inflammatory stimuli upregulate HIF-1α in M1 microglia, shifting metabolism to glycolysis. This elevates ROS production, chronically activating the NLRP3 inflammasome (Tian et al. 2025b; Rosencrans et al. 2025). Normally, PINK1/Parkin-mediated mitophagy clears damaged mitochondria (Zhang et al. 2025a; Zhou et al. 2025). However, AD and PD severely impair this process; the resulting accumulation of defective mitochondria causes cytosolic mtDNA efflux, engaging the cGAS-STING pathway to amplify oxidative stress and microglial activation in a self-reinforcing cycle (Li et al. 2025a; Quan et al. 2025).

### Novel immune signaling

#### The cGAS-STING pathway

**Fig. 4:**
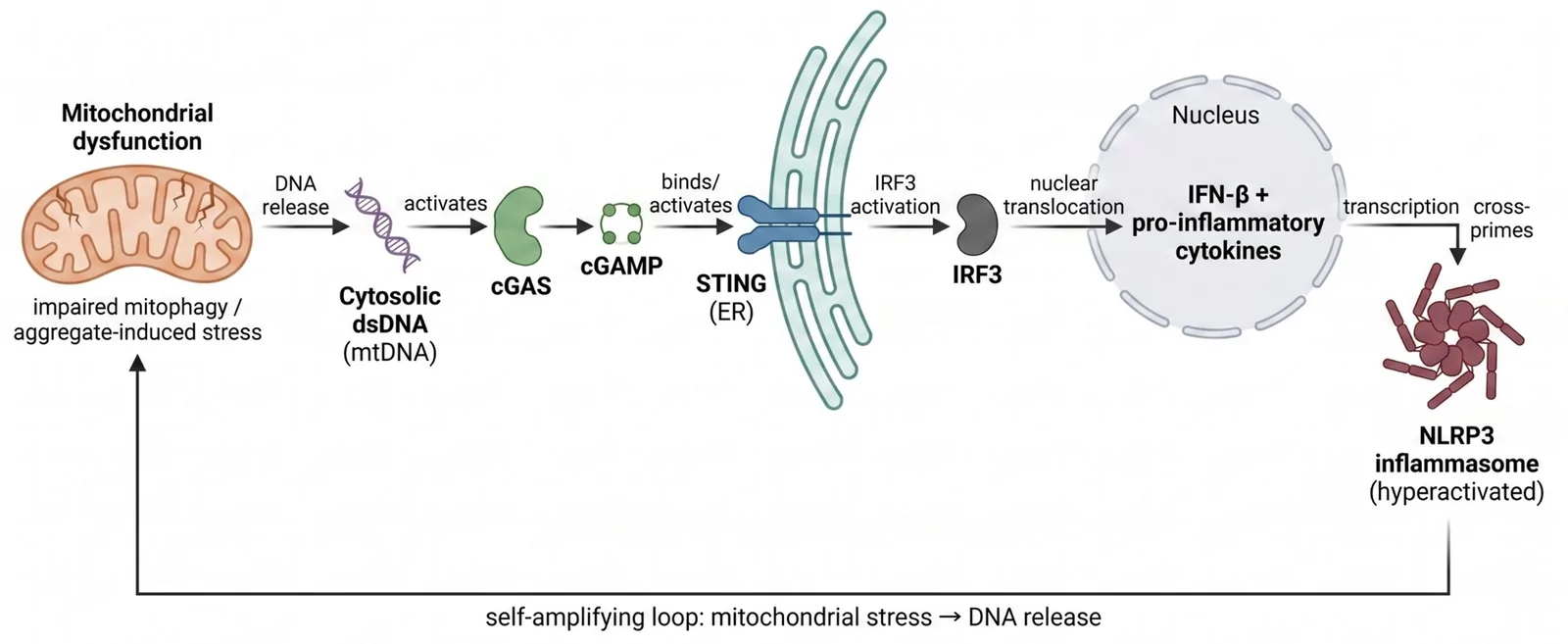
Crosstalk in Neurodegeneration: cGAS-STING Signaling and the NLRP3 Inflammasome. This schematic illustrates how cytosolic DNA released from impaired mitophagy or aggregate-induced cellular stress activates the cGAS–STING pathway. Cytosolic DNA sensing by cGAS leads to cGAMP production and subsequent STING activation on the endoplasmic reticulum, promoting IRF3 nuclear translocation and type I IFN-β production. STING signaling also enhances pro-inflammatory cytokine expression, contributing to NLRP3 inflammasome priming and activation. The diagram highlights the reciprocal crosstalk between cGAS–STING and NLRP3 pathways as a convergent driver of chronic neuroinflammation. Nano-immunomodulatory strategies targeting this axis, including co-delivery of STING inhibitors and NLRP3 antagonists, represent potential approaches to disrupt this self-amplifying inflammatory network. Abbreviations: cGAS–STING: Cyclic GMP-AMP Synthase Stimulator of Interferon Genes, IFN-β: Interferon-beta, IRF3: Interferon Regulatory Factor 3, NLRP3: NOD-, LRR- and pyrin domain-containing protein 3

This cascade mechanistically links mitochondrial dysfunction to NLRP3 hyperactivation. Cytosolic DNA from impaired mitophagy or aggregate-induced cellular stress activates cyclic GMP-AMP synthase (cGAS), which synthesizes cGAMP to bind endoplasmic reticulum-resident STING (Jauhari et al. 2026; Zhao et al. 2026). This engagement induces IRF3 nuclear translocation, driving type I interferon (IFN-β) and pro-inflammatory cytokine transcription (Qi et al. 2025; Wang et al. 2025b). This continuous signaling cross-primes the NLRP3 inflammasome, establishing a self-amplifying neurotoxic loop (Eskandari-Sedighi et al. 2023) (Fig. 4).

#### The CD33-TREM2 axis

**Fig. 5:**
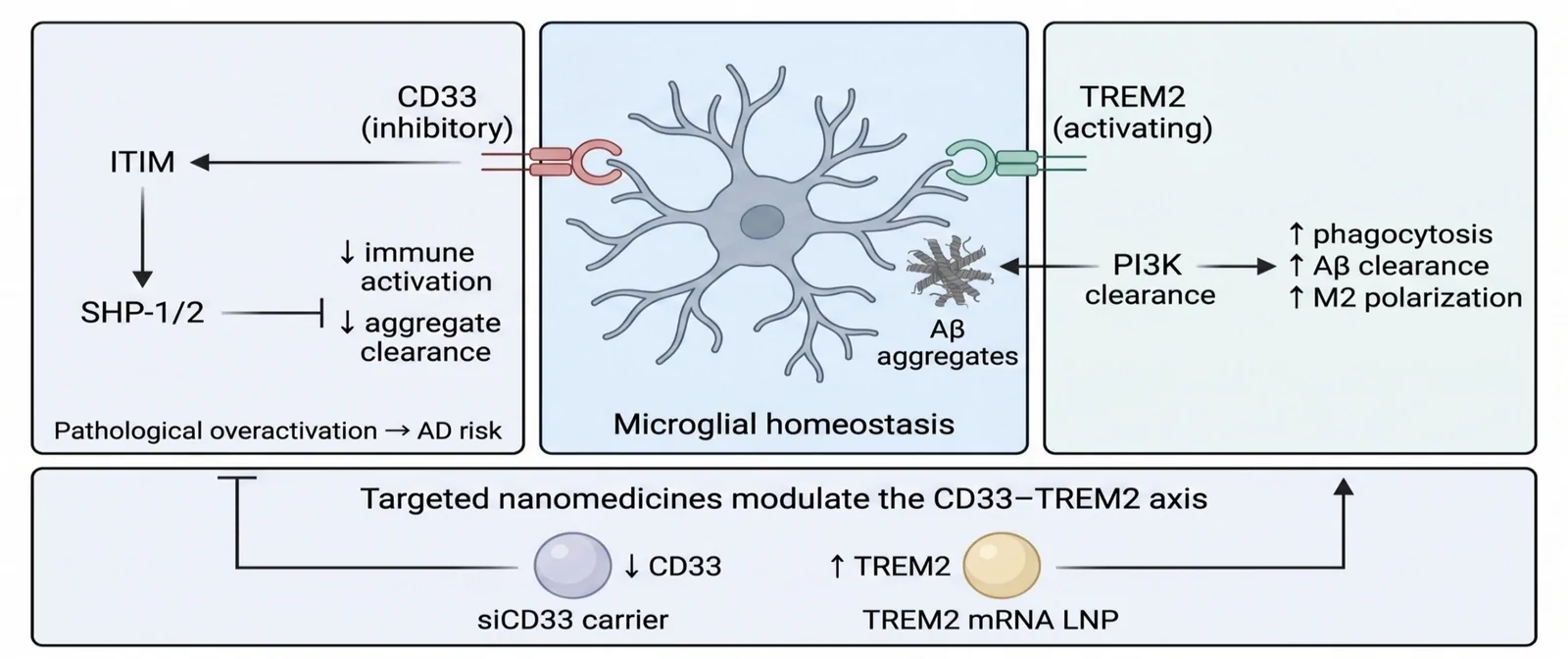
Restoring Microglial Homeostasis: The Role of the CD33-TREM2 Axis and Nanomedicine. This schematic illustrates the opposing regulatory roles of CD33 and TREM2 in controlling microglial function. CD33 activation recruits SHP-1/2 phosphatases through its immunoreceptor tyrosine-based inhibitory motifs (ITIMs), suppressing phagocytic activity and reducing pathological aggregate clearance, a mechanism associated with increased AD susceptibility. In contrast, TREM2 signaling through PI3K promotes Aβ uptake, supports metabolic adaptation, and favors protective microglial functions. The figure further highlights nanomedicine-based strategies, including lipid nanoparticles delivering TREM2 mRNA and siCD33 cargoes, as potential approaches to rebalance this regulatory axis, restore microglial homeostasis, and reduce neuroinflammatory burden in AD and PD. Abbreviations: AD: Alzheimer's Disease, CD33: Cluster of Differentiation 33, ITIM: Immunoreceptor Tyrosine-based Inhibitory Motif, mRNA: Messenger Ribonucleic Acid, PI3K: Phosphoinositide 3-kinase, SHP-1/2: Src Homology region 2 domain-containing Phosphatase−1/2, TREM2: Triggering Receptor Expressed on Myeloid cells 2

**Fig. 6:**
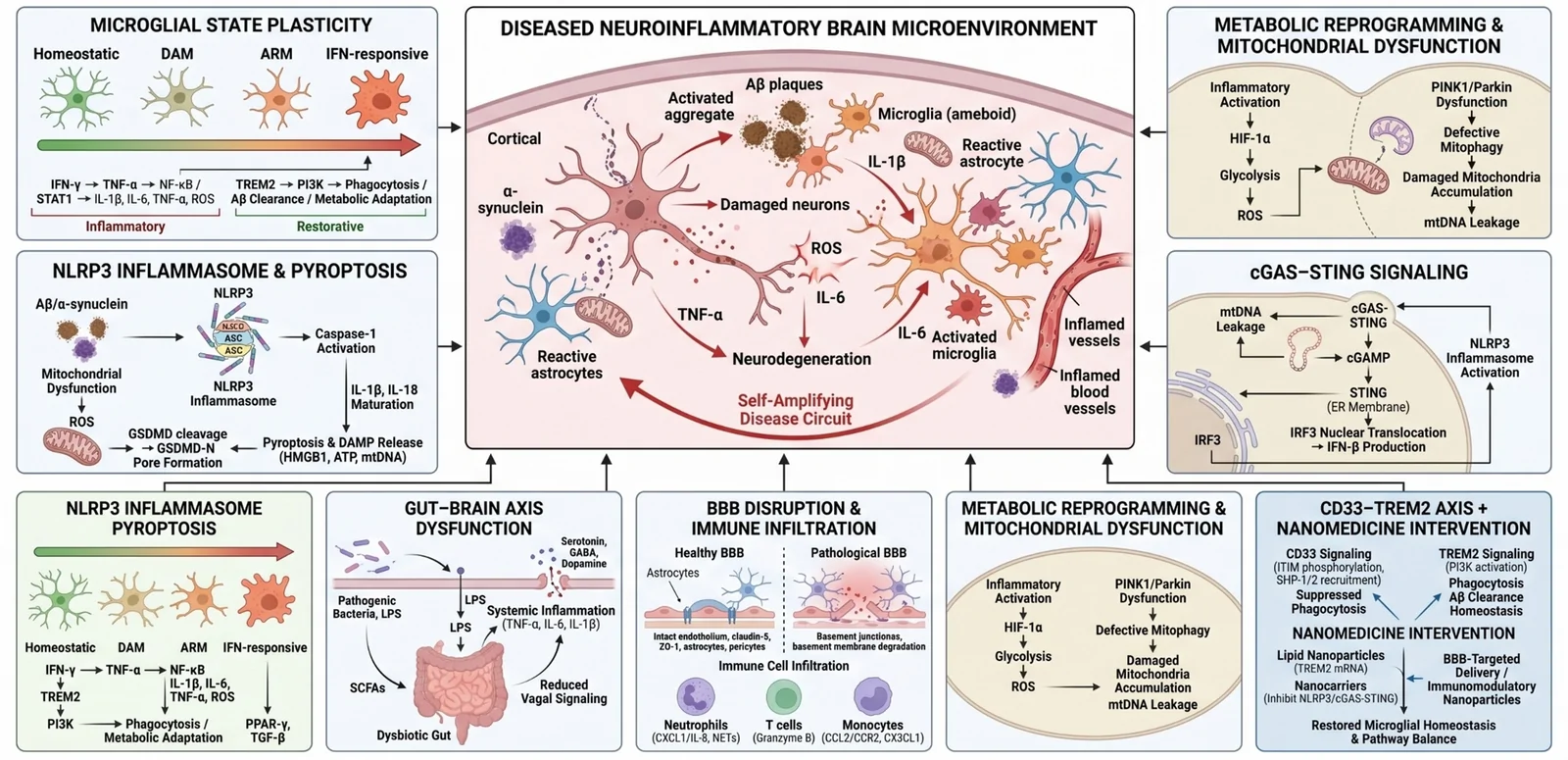
Integrated molecular and cellular mechanisms underlying neuroinflammation in AD and PD, illustrating the interaction between microglial dysfunction, inflammasome activation, mitochondrial impairment, neurovascular alterations, peripheral immune recruitment, and emerging nanomedicine-based interventions. The schematic depicts the neuroimmune landscape, where pathological protein aggregates including Aβ plaques and α-synuclein initiate neuronal stress and activate surrounding immune and glial cells. Microglia transition from homeostatic and repair phenotypes to DAM, activated response ARM, and interferon-responsive states. IFN-γ/TNF-α-driven NF-κB and STAT1 signaling promotes cytokine release, oxidative stress, and inflammation, whereas TREM2–PI3K, PPAR-γ, and TGF-β pathways support phagocytosis, metabolic adaptation, and microglial homeostasis. Aβ/α-synuclein accumulation, mitochondrial dysfunction, and ROS trigger NLRP3 inflammasome assembly. Subsequent activation of the NLRP3–ASC–caspase−1 complex promotes IL-1β and IL-18 maturation, GSDMD cleavage, and pyroptosis. This releases inflammatory DAMPs including HMGB1, extracellular ATP, and mtDNA further reinforcing neuroinflammation. Metabolic and mitochondrial alterations drive inflammatory microglial reprogramming toward glycolysis via HIF-1α activation, alongside increased ROS, impaired PINK1/Parkin-mediated mitophagy, mitochondrial damage, and mtDNA leakage. Cytosolic mtDNA activates the cGAS–STING pathway, inducing cGAMP production, STING activation, IRF3 nuclear translocation, and IFN-β-mediated signaling. This cascade converges with NLRP3 activation to establish a self-amplifying inflammatory loop. Systemic contributors to neurodegeneration include gut–brain axis dysfunction and BBB disruption. Gut dysbiosis increases pathogenic bacterial products like LPS and reduces protective SCFAs, elevating systemic cytokines and altering neuroimmune communication. Concurrently, BBB impairment driven by endothelial dysfunction, tight-junction loss, extracellular matrix degradation, and astrocytic dysfunction facilitates the infiltration of peripheral immune cells (neutrophils, T cells, and monocyte-derived macrophages) to sustain inflammation. Finally, the CD33–TREM2 regulatory axis and nanomedicine approaches offer strategies to restore microglial balance. While CD33-mediated inhibitory signaling reduces phagocytosis and aggregate clearance, TREM2 activation promotes protective microglial functions. Targeted nanoplatforms including lipid nanoparticles, RNA delivery systems, and immunomodulatory nanocarriers can selectively regulate inflammatory pathways, enhance microglial homeostasis, and restore neuroimmune equilibrium. Abbreviations: AD: Alzheimer’s Disease; PD: Parkinson’s Disease; Aβ: amyloid-β; DAM: Disease-Associated Microglia; ARM: Activated Response Microglia, ROS: Reactive Oxygen Species, NLRP3: NOD-, LRR- and Pyrin Domain-Containing Protein 3, ASC: Apoptosis-Associated Speck-like protein containing a CARD, GSDMD: Gasdermin D, DAMPs: Damage-Associated Molecular Patterns, BBB: Blood–Brain Barrier, LPS: Lipopolysaccharide, SCFAs: Short-Chain Fatty Acids, mtDNA: mitochondrial DNA, cGAS: Cyclic GMP–AMP Synthase, STING: Stimulator of Interferon Genes, IRF3: Interferon Regulatory Factor 3, CD33: Cluster of differentiation 33, TREM2: Triggering Receptor Expressed on Myeloid Cells 2

Microglial function is strongly shaped by the inhibitory CD33 and activating TREM2 receptors in AD, whereas PD more prominently involves microglial responses to α-synuclein, altered dopamine metabolism, and region-specific innate immune activation (Satoh et al. 2025; Abdulkhaliq et al. 2026a). CD33 binding triggers an ITIM motif (recruiting SHP-1/2 phosphatases) to suppress immune activation; its pathological overactivation limits aggregate clearance and correlates with AD risk (Che et al. 2026; Lin et al. 2025a). Conversely, TREM2 signaling via the PI3K pathway promotes phagocytosis, Aβ clearance, and M2 polarization (Huang et al. 2026a; Liu et al. 2014). Modulating this axis using targeted nanomedicines such as TREM2 mRNA-delivering lipid nanoparticles or siCD33-loaded carriers represents a precise strategy to restore microglial homeostasis (Fig. 5). The complex interplay between microglial dysregulation, inflammasome activation, mitochondrial dysfunction, gut-brain axis disruption, and peripheral immune infiltration establishes a self-amplifying neuroinflammatory network that drives neurodegenerative progression. The major molecular and cellular interactions underlying this inflammatory landscape and potential intervention points for nanomedicine-based modulation are summarized in Fig. 6.

## Latest advances in nano-immunomodulator design and function

Nano-immunomodulatory strategies for neurodegenerative diseases encompass diverse but interconnected platform classes with distinct therapeutic mechanisms. Intrinsically active nanomaterials, including polydopamine nanoparticles and nanozymes, reduce oxidative stress and modulate redox-sensitive inflammatory pathways. Targeted nanocarriers, such as nanobody-functionalized systems and engineered exosomes, enhance BBB penetration and enable cell- or pathology-specific delivery. Drug-loaded and stimuli-responsive nanoplatforms provide controlled release of immunomodulatory cargos in response to disease-associated signals, including ROS, pH changes, and protease activity.

Precision pathway-interceptor systems enable targeted regulation of key inflammatory networks, particularly NLRP3 inflammasome and cGAS–STING signaling, while nucleic acid delivery platforms, especially lipid nanoparticles (LNPs), support microglial reprogramming through mRNA, siRNA, and gene-editing approaches. Collectively, these advances extend nanomedicine beyond general inflammatory suppression toward integrated strategies combining antioxidant activity, targeted delivery, pathway-specific modulation, and long-term immune regulation.

### Direct immunomodulatory nanomaterials

Building on the molecular targets discussed in Sect. "Neuroinflammation mechanisms and pathways", this section examines nanoplatforms designed to regulate specific pathological mechanisms, with Table 5 summarizing the corresponding mechanism-platform relationships. Nanomaterials with intrinsic anti-inflammatory activity represent a promising approach for chronic neuroinflammation in AD and PD. Rather than promoting a simplified M1-to-M2 transition, these platforms may regulate microglial state plasticity by suppressing persistent inflammatory programs while supporting homeostatic, phagocytic, and repair-associated functions. Unlike passive delivery systems, intrinsically active nanomaterials directly reduce oxidative stress, modulate pro-inflammatory signaling, and support neuronal preservation without requiring encapsulated therapeutic cargos. Representative examples include polydopamine nanoparticles (PDNPs) and biomimetic nanozymes.

#### Polydopamine nanoparticles

Polydopamine nanoparticles (PDNPs) exhibit intrinsic anti-inflammatory activity primarily through the redox properties of polymerized polydopamine (Ryu et al. 2018). Their abundant catechol and quinone groups provide strong radical-scavenging and electron-donating capacities, enabling ROS neutralization and reduction of oxidative stress (Wang et al. 2026a). By limiting ROS accumulation, PDNPs can attenuate oxidative stress-driven inflammatory signaling and protect CNS cells from oxidative damage (Wang et al. 2026a).

Beyond their antioxidant effects, PDNPs have been reported to suppress NF-κB signaling and modulate pro-inflammatory microglial responses, potentially favoring more homeostatic or repair-associated states within the broader activation continuum (Duță et al. 2025). This regulation may reduce inflammatory mediators, including IL-1β and TNF-α, thereby improving neuroimmune balance [64/127,65/128] (Table 3).

**Table 3:** . Design strategies, functional properties, and therapeutic mechanisms of nano-immunomodulators

Class / paradigm	Nanomaterial / platform	Structural & functional properties	biological & therapeutic mechanisms	References
Direct immunomodulatory nanomaterials	Polydopamine Nanoparticles (PDNPs)	Robust ROS scavenging capabilities; do not require encapsulated payloads	Attenuates oxidative stress; prevents downstream inflammatory cascade activation; protects CNS cells	Huang et al. (2026a), Duan et al. (2026)
		Modulator of inflammatory signaling	Suppresses NF-κB signaling; inhibits microglial M1 activation; promotes shift toward the neuroprotective M2 phenotype	Huang et al. (2026a), Duan et al. (2026)
		Cytokine regulator	Reduces pro-inflammatory cytokine release (e.g., IL-1β, TNF-α); restores CNS immune homeostasis	Huang et al. (2026a), Duan et al. (2026)
	Biomimetic Nanozymes	Replicates natural antioxidant enzymes (e.g., SOD, CAT)	Efficiently scavenges superoxide radicals and H₂O₂	Godugu et al. (2025), Amniouel et al. (2025), Ma et al. (2025b)
		Modulator of mitochondrial function	Promotes mitophagy; prevents cytosolic release of mtDNA and DAMPs; prevents activation of innate immune sensors (e.g., cGAS-STING pathway); interrupts self-amplifying neuroinflammation	Godugu et al. (2025), Amniouel et al. (2025), Ma et al. (2025b)
Targeted delivery platforms	Nanobody (sdAb)-based systems	Compact (~ 15 kDa) camelid-derived fragments	Facilitates superior BBB traversal and tissue penetration	Lafon et al. (2025, Naeem et al. (2024)
		Lacks an Fc region	Avoids complement activation and immunogenicity	Tsitokana et al. (2023)
		Ligands recognizing disease-specific structures (e.g., Aβ or α-synuclein) without steric hindrance	Conjugation to nanocarriers concentrates therapeutics at pathological sites; enables precise intervention with minimal systemic exposure	Liu et al. (2025c)
	Exosome-Based Nanocarriers	Natural, cell-derived vectors featuring a lipid bilayer and surface functionalization	Provides high biocompatibility and BBB crossing; encapsulates diverse cargoes; directs delivery to specific brain cells	Huang et al. (2026b), Onohuean et al. (2025), Évora et al. (2025), Feng et al. (2025)
		Engineered carriers of miRNAs	Silences pro-inflammatory microglial genes; suppresses NLRP3 inflammasome activation; restores immune balance	An et al. (2025), Shahlaei et al. (2025)
		Dual-delivery system	Co-delivers pathology inhibitors (tau/Aβ targets) and neurotrophins to address multiple neurodegenerative mechanisms concurrently	Abdulkhaliq et al. (2026b), Kudpaje et al. (2025), Ke et al. (2026)
Nanotheranostics	Diagnostic & therapeutic systems	Combines diagnostic imaging and targeted therapy	Overcomes poor specificity and CNS penetration of conventional treatments	Zhao et al. (2025a), Sun et al. (2025b))
		Integrates MRI contrast agents (iron oxide, gadolinium) or fluorescent probes	Enables real-time visualization of neuroinflammation	Kochman et al. (2026), Araujo et al. (2026), Prasanth et al. (2025)
		Employs stimulus-responsive drug release triggered by microenvironmental cues	Achieves targeted release of therapeutic agents	Ballance et al. (2019)
		Features pH-sensitive coatings, ROS-sensitive linkers, or enzymatic triggers (e.g., MMPs, cathepsins)	Localizes release of immunomodulators (e.g., MCC950) specifically in acidic tissues, high oxidative stress sites, or active pathological sites	Ballance et al. (2019)
		Spatiotemporally controlled design	Enhances therapeutic efficacy while reducing systemic side effects	Ballance et al. (2019)

*Aβ* Amyloid beta, *AD* Alzheimer's disease, *BBB* Blood–brain barrier, *CAT* Catalase, *cGAS* Cyclic GMP-AMP synthase, *CNS* Central nervous system, *DAMPs* Damage-associated molecular patterns, *Fc* Fragment crystallizable, *H₂O₂* Hydrogen peroxide, *IL-1β* Interleukin-1 beta, *miRNAs* MicroRNAs, *MMPs* Matrix metalloproteinases, *MRI* Magnetic resonance imaging, *mtDNA* Mitochondrial DNA, *NF-κB* Nuclear factor kappa-light-chain-enhancer of activated B cells, *NLRP3* NLR family pyrin domain containing 3, *PD* Parkinson's disease, *PDNPs* Polydopamine nanoparticles, *ROS* Reactive oxygen species, *sdAb* Single-domain antibody, *SOD* Superoxide dismutase, *STING* Stimulator of interferon genes, *TNF-α* Tumor necrosis factor alpha

Preclinical studies indicate that PDNP-based formulations can reduce ROS levels, attenuate microglial activation, and decrease inflammatory cytokine production in models of CNS injury and neurodegeneration, supporting their potential as redox-modulating nanotherapeutics (Ceccarelli et al. 2025).

PDNP surface chemistry may also facilitate interactions with inflammatory microenvironments and indirectly reduce inflammasome priming and downstream cytokine amplification (Wang et al. 2026b). These effects arise from the nanoparticle structure and surface properties of polymerized polydopamine rather than unpolymerized dopamine, which lacks the architectural features responsible for these immunomodulatory functions (Golchin et al. 2017).

#### Biomimetic nanozymes

Biomimetic nanozymes are engineered nanomaterials that mimic the catalytic activities of natural enzymes under physiological conditions (Chen et al. 2026). In neuroinflammatory settings, these platforms are primarily designed to replicate antioxidant enzymes, particularly superoxide dismutase (SOD) and catalase (CAT), enabling catalytic removal of superoxide radicals and H_2_O_2_ (Godugu et al. 2025; Amniouel et al. 2025; Ma et al. 2025b). Through sustained ROS scavenging, nanozymes can reduce oxidative stress and preserve mitochondrial function (Xia et al. 2025).

Beyond redox regulation, certain nanozyme platforms have been reported to enhance mitophagy and promote clearance of damaged mitochondria, thereby limiting cytosolic accumulation of mitochondrial DNA (mtDNA) and other damage-associated molecular patterns (DAMPs) that activate innate immune pathways such as cGAS–STING (Xia et al. 2025). By simultaneously targeting oxidative stress and mitochondrial dysfunction, these systems may attenuate self-amplifying inflammatory circuits and promote less inflammatory, more phagocytic microglial states (Zha et al. 2024) (Table 3).

Preclinical studies have shown that biomimetic nanozymes provide catalytic ROS regulation, preserve mitochondrial function, reduce mtDNA-associated innate immune activation, and improve neuroprotective outcomes in inflammatory and neurodegenerative models (Xia et al. 2025). Collectively, these findings support their potential as enzyme-mimetic nanotherapeutics targeting interconnected oxidative and mitochondrial mechanisms underlying neuroinflammation (Zha et al. 2024).

### Targeted delivery platforms

The BBB remains a major obstacle to effective therapeutic delivery in AD and PD (Serrano et al. 2025). Targeted nanomedicine platforms, including nanobody-functionalized systems and engineered exosomes, are being developed to improve CNS delivery, enhance spatial selectivity, and reduce systemic off-target exposure (Boado 2025).

Nanocarriers can interact with or cross the BBB through several complementary mechanisms (Serrano et al. 2025). Receptor-mediated transcytosis (RMT) exploits endogenous endothelial receptors, including the transferrin receptor, low-density lipoprotein receptor-related protein 1 (LRP1), and insulin receptor (Mehdizadeh et al. 2025). Functionalization with specific ligands, peptides, or antibody fragments facilitates receptor binding, endothelial internalization, vesicular trafficking, and BBB transport (Liu et al. 2025c).

Adsorptive-mediated transcytosis (AMT) relies on electrostatic interactions between positively charged nanocarrier surfaces and negatively charged endothelial glycocalyx components, promoting nonspecific endocytosis and transcellular transport (Li et al. 2024). However, excessive surface charge may reduce targeting specificity, increase protein adsorption, and contribute to vascular toxicity (Rautio et al. 2013).

Carrier- or transporter-mediated strategies exploit endogenous nutrient transport systems, such as those for glucose and amino acids, by incorporating transporter-recognized substrates or mimetic ligands into nanocarrier designs (Mehdizadeh et al. 2025). Their effectiveness depends on transporter specificity, competition with endogenous substrates, and preservation of ligand recognition after nanocarrier modification (Choi et al. 2012).

Cell-mediated delivery, often termed a “Trojan horse” strategy, uses migratory cells such as monocytes, macrophages, or stem cells to facilitate nanomedicine transport across the BBB (Mohammed et al. 2025). Although potentially advantageous under inflammatory conditions that increase immune-cell trafficking, this approach is limited by variable biodistribution, inadequate control of cellular migration, and potential effects on inflammatory recruitment (Jo et al. 2015).

BBB-targeted delivery efficiency is influenced by nanocarrier physicochemical properties, including size, shape, surface charge, ligand density, colloidal stability, and protein-corona composition (Ceña and Játiva 2018).

Although optimized surface engineering can improve BBB interactions, no universal size or charge parameters ensure successful transport across different platforms (Wang et al. 2025c). Delivery outcomes are also affected by ligand–receptor interactions, disease-associated BBB alterations, species differences, and the experimental model used (Lee et al. 2026).

Preclinical evaluation of BBB-targeted nanomedicines commonly assesses brain-to-blood distribution, endothelial uptake, transendothelial transport, parenchymal localization, and uptake by target CNS cells, together with BBB integrity and therapeutic efficacy (Zhang et al. 2025c).

Relevant outcomes include modulation of inflammatory cytokines, enhanced clearance of pathological aggregates, and improvements in functional or behavioral measures (Zeng et al. 2026).

Despite substantial progress, clinical translation remains limited by interspecies differences, disease-dependent BBB heterogeneity, peripheral organ accumulation, and the lack of standardized methods for quantifying brain delivery (Lee et al. 2026). Major receptors and transport systems used for BBB targeting, along with representative ligands and preclinical evidence, are summarized in Table 4.

**Table 4:** Mechanisms of nanocarrier transport across the BBB

Transport strategy	Mechanism of action	Key facilitators & substrates	Limitations & dependencies	References
RMT	Exploits endogenous endothelial receptors to facilitate receptor binding, endothelial internalization, vesicular trafficking, and BBB transport	Receptors: Transferrin receptor, LRP1, insulin receptor Functionalization: Specific ligands, peptides, or antibody fragments	–	Mehdizadeh et al. (2025), Liu et al. (2025c)
AMT	Promotes nonspecific endocytosis and transcellular transport via electrostatic interactions	Positively charged nanocarrier surfaces and negatively charged endothelial glycocalyx components	Excessive surface charge may reduce targeting specificity, increase protein adsorption, and contribute to vascular toxicity	Li et al. (2024), Rautio et al. (2013)
Carrier- or transporter-mediated delivery	Exploits endogenous nutrient transport systems by incorporating mimetic ligands or recognized substrates into nanocarrier designs	Nutrient transport systems (e.g., for glucose and amino acids)	Effectiveness depends on transporter specificity, competition with endogenous substrates, and preservation of ligand recognition post-modification	Mehdizadeh et al. (2025), Choi et al. (2012)
Cell-mediated delivery (“trojan horse”)	Utilizes migratory cells to facilitate nanomedicine transport; potentially advantageous under inflammatory conditions that increase immune-cell trafficking	Monocytes, macrophages, or stem cells	Limited by variable biodistribution, inadequate control of cellular migration, and potential effects on natural inflammatory recruitment	Mohammed et al. (2025), Jo et al. (2015)

*AMT* Adsorptive-Mediated Transcytosis, *BBB* Blood–Brain Barrier, *LRP1* Low-Density Lipoprotein Receptor-Related Protein 1, *RMT* Receptor-Mediated Transcytosis

#### Nanobody (sdAb)-based systems

Nanobodies (single-domain antibodies) are compact (~ 15 kDa) camelid-derived antibody fragments that offer enhanced tissue penetration and potential advantages for BBB delivery (Lafon et al. 2025; Naeem et al. 2024). Their lack of an Fc region reduces Fc-mediated immune activation and may limit immunogenicity-related concerns (Tsitokana et al. 2023). These small binding domains can selectively recognize disease-associated targets, including Aβ and α-synuclein aggregates, with reduced steric constraints compared with conventional antibodies.

When conjugated to nanocarriers, nanobodies can improve accumulation of therapeutic cargos at pathological sites, enabling more selective intervention while potentially reducing systemic exposure (Liu et al. 2025c) (Table 3). In human BBB models and preclinical neurodegenerative disease studies, nanobody-functionalized platforms have demonstrated enhanced receptor-mediated transcytosis, increased brain accumulation, and selective targeting of pathological protein conformations, supporting their role as promising BBB-shuttling ligands for precision CNS delivery (Esparza et al. 2023; Jootar et al. 2026; Sun et al. 2025a).

#### Exosome-based nanocarriers

Natural, cell-derived exosomes offer high biocompatibility and BBB-crossing capabilities, making them ideal CNS drug vectors (Huang et al. 2026b; Onohuean et al. 2025). Their lipid bilayer encapsulates diverse cargoes (miRNAs, small molecules, neurotrophins), while surface functionalization directs them to specific brain cells (Évora et al. 2025; Feng et al. 2025). Engineered exosomes can deliver miRNAs to silence pro-inflammatory microglial genes and suppress NLRP3 inflammasome activation, restoring immune balance (An et al. 2025; Shahlaei et al. 2025). They can also simultaneously co-deliver pathology inhibitors (targeting tau or Aβ) and neurotrophins to address multiple neurodegenerative mechanisms concurrently (Abdulkhaliq et al. 2026b; Kudpaje et al. 2025; Ke et al. 2026) (Table 3).

Preclinical exosome studies have shown that engineered vesicles can increase CNS delivery of miRNA and protein cargoes, suppress microglial inflammatory signaling, and improve disease-related phenotypes in experimental AD and PD models, underscoring their translational value as naturally derived drug carriers (Huang et al. 2026b; Salarpour et al. 2025; Wang et al. 2024).

### Nanotheranostics

Nanotheranostic platforms integrate diagnostic imaging and targeted therapy to overcome limitations of conventional treatments, including poor specificity and limited CNS penetration (Zhao et al. 2025a; Sun et al. 2025b). Diagnostic components commonly include MRI contrast agents, such as iron oxide and gadolinium-based materials, or fluorescent probes for real-time visualization of neuroinflammatory processes (Kochman et al. 2026; Araujo et al. 2026; Prasanth et al. 2025). These systems can detect disease-associated features, including Aβ plaques, tau aggregates, and α-synuclein pathology, as well as microenvironmental signals such as local acidosis, elevated ROS levels, and increased matrix metalloproteinase (MMP) activity, enabling spatial and temporal characterization of pathological regions (Zhao et al. 2025a; Sun et al. 2025b; Araujo et al. 2026; Prasanth et al. 2025; Ballance et al. 2019).

Therapeutic components are often engineered for stimulus-responsive release triggered by disease-specific cues (Liu et al. 2026a). pH-sensitive coatings promote cargo release in acidic environments, ROS-responsive linkers activate under oxidative stress, and enzyme-responsive systems using proteases such as MMPs or cathepsins enable localized delivery at active pathological sites (Zhang et al. 2023a, 2023b; Guo et al. 2024b). These strategies improve spatial and temporal control of therapeutic exposure, potentially enhancing efficacy while reducing systemic toxicity (Zhang et al. 2023a).

By combining diagnostic monitoring with microenvironment-responsive drug release, nanotheranostic platforms enable imaging-guided anti-inflammatory intervention (Zhao et al. 2025b). Pathological signals used for lesion detection can also serve as activation triggers, allowing localized delivery of immunomodulatory agents such as MCC950 and other neuroprotective cargos while minimizing off-target effects (Tian et al. 2025c). However, most nanotheranostic systems remain at an early preclinical stage, with integrated imaging and therapeutic performance demonstrated mainly in rodent and ex vivo models rather than human CNS platforms (Zhao et al. 2025b) (Table 3).

Preclinical studies have shown that nanotheranostic approaches can combine real-time visualization of pathological lesions with localized therapeutic release, providing proof-of-concept for spatiotemporally controlled intervention in neuroinflammatory disease models (Zhao et al. 2025b).

### Precision pathway interceptors in neurodegenerative diseases

Precision pathway interceptors are engineered nanomaterials designed to disrupt self-amplifying molecular circuits that sustain chronic neuroinflammation by targeting multiple pathological nodes simultaneously (Xie et al. 2023). One example is the AD Aβ–STING axis, in which Aβ accumulation can activate microglial cGAS–STING signaling, impair phagocytic function, and reinforce pro-inflammatory microglial states (Chung et al. 2024). The ASSIST system (Aβ-targeting, STING-inhibiting, and immunomodulatory nanomaterials) represents a proof-of-concept approach within this category (Hettiarachchi et al. 2019).

ASSIST nanoparticles have been reported to target Aβ plaques while co-delivering components that suppress STING activation (Mitchell et al. 2021). This dual-action strategy may reduce inflammatory cytokine production and promote a more balanced microglial phenotype; however, its therapeutic potential remains limited by insufficient in vivo validation and clinical evidence (Chung et al. 2024).

Although these platforms demonstrate the potential of precision nano-immunomodulation, their translation requires assessment beyond therapeutic efficacy, including manufacturing scalability, biological complexity, safety, and regulatory considerations (Topal et al. 2020). Preclinical studies of ASSIST-like and related multi-target nanointerceptors have shown reduced Aβ-associated inflammatory signaling, enhanced phagocytic activity, and improved microglial functional balance, supporting the feasibility of simultaneously targeting pathological aggregation and innate immune activation (Lin et al. 2025b; Ren et al. 2025; Younis et al. 2022).

### Limitations and translational challenges of current NIM platforms

Despite their mechanistic advantages, current NIM platforms face substantial platform-specific barriers to clinical translation (Gkogkos et al. 2023). Polydopamine nanoparticles and biomimetic nanozymes provide intrinsic catalytic and antioxidant activities; however, their synthesis is highly sensitive to reaction conditions, including pH, oxygen availability, and precursor concentration, which can affect particle size, surface properties, and biological activity (Battaglini et al. 2025). Their reactive surfaces may also promote protein corona formation in biological fluids, altering targeting efficiency, biodistribution, and ROS-scavenging performance (Sun et al. 2024; Mei et al. 2025). Moreover, the long-term biodegradation and clearance of carbon- and metal-based nanozyme systems remain insufficiently characterized, raising concerns about accumulation after repeated administration (Toader et al. 2024; Zheng et al. 2022).

Nanobody-based platforms offer high targeting specificity and reduced Fc-mediated immune activation but may still induce anti-drug antibody responses after repeated exposure (Ackaert et al. 2021; Pauw et al. 2023; Xenaki et al. 2021). Their small size also promotes rapid renal clearance, necessitating half-life extension strategies that increase design complexity (Ackaert et al. 2021; Peng et al. 2020; Zeng et al. 2023). Exosome-based carriers provide high biocompatibility but are limited by donor-cell variability, inconsistent cargo loading, and the lack of standardized production and quality-control procedures (Koh et al. 2023; Palakurthi et al. 2024). Their complex biological composition further complicates regulatory classification and manufacturing standardization (Lankoff et al. 2024).

Nanotheranostic systems face additional challenges because their multifunctional architectures often combine imaging agents, targeting ligands, therapeutic cargos, and responsive components (Shanahan et al. 2025; Crist et al. 2021). This complexity increases manufacturing demands, cost, and batch variability, while the long-term safety of retained imaging materials, including metal-based contrast agents, requires further evaluation (Li et al. 2026). Similarly, precision pathway interceptors such as ASSIST-like systems remain largely confined to early preclinical validation (Xie et al. 2023; Joseph and Nance 2022; Stucchi et al. 2026). Their multi-component design adds regulatory complexity because each functional element may require independent assessment of safety, stability, and pharmacological performance (Zhang et al. 2025d) (Table 5).

**Table 5:** Integrated map of molecular targets and corresponding nano-immunomodulator platforms

Molecular Target/Pathway	Pathological role in AD/PD	Corresponding NIM platform(s)	Mechanism of nanotherapeutic intervention	References
NF-κB/STAT1 (M1 microglial polarization)	Drives pro-inflammatory IL-1β, IL-6, TNF-α, ROS, NO release; exacerbates neuronal apoptosis	Polydopamine nanoparticles (PDNPs); metabolic modulator-delivering nanocarriers	Suppresses NF-κB signaling, inhibits M1 activation, shifts microglia toward neuroprotective M2 phenotype	Varsamos et al. (2025), Shi et al. (2026), Guo et al. (2024a), Masuda et al. (2020), Huang et al. (2026a), Duță et al. (2025), Duan et al. (2026)
NLRP3 inflammasome	Matures IL-1β/IL-18, drives gasdermin-D pyroptosis, self-amplifying neurotoxic cycle	Biomimetic nanozymes; nanotheranostics with MCC950 stimulus-responsive release; nanobody-drug conjugates	ROS scavenging and mitophagy promotion prevent mtDNA/DAMP release; stimulus-responsive delivery of NLRP3 antagonists (e.g., MCC950) at inflammasome priming sites	Xu et al. (2025b), Kayagaki et al. (2015), Ding et al. (2016), Kaczmarek et al. (2013), Bours et al. (2006), Godugu et al. (2025), Amniouel et al. (2025), Ma et al. (2025b), Xia et al. (2025), Zha et al. (2024), Kochman et al. (2026), Zhao et al. (2025a), (2025b), Sun et al. (2025b), Araujo et al. (2026), Prasanth et al. (2025), Ballance et al. (2019), Liu et al. (2026a), Zhang et al. (2023a), (2023b), Guo et al. (2024b), Tian et al. (2025c), Sela et al. (2025), Rafiei et al. (2025), Feiten et al. (2026), Liao et al. (2025), Han et al. (2024)
cGAS-STING pathway	Cytosolic DNA sensing drives IRF3-mediated type I interferon production, cross-primes NLRP3	Biomimetic nanozymes; precision pathway interceptors (e.g., ASSIST system)	Prevents cytosolic mtDNA/DAMP accumulation; co-delivers STING-inhibiting payloads alongside Aβ-clearance agents to dismantle the Aβ-STING cycle	Jauhari et al. (2026), Zhao et al. (2026), Qi et al. (2025), Wang et al. (2025b), Eskandari-Sedighi et al. (2023) Chen et al. (2026), Godugu et al. (2025), Amniouel et al. (2025), Ma et al. (2025b), Xia et al. (2025), Xie et al. (2023), Chung et al. (2024), Hettiarachchi et al. (2019), Mitchell et al. (2021), Topal et al. (2020), Lin et al. (2025b), Ren et al. (2025), Younis et al. (2022)
PINK1/Parkin-mediated mitophagy	Impaired clearance of damaged mitochondria causes mtDNA efflux, engaging cGAS-STING and amplifying oxidative stress	Biomimetic nanozymes; metabolite-sensing nanoplatforms (emerging)	Enhances mitophagic clearance of defective mitochondria, interrupting the ROS-mtDNA-cGAS-STING amplification loop	Zhang et al. (2025a), Huang et al. (2024), Nirakis et al. (2025), Tian et al. (2025b),, Rosencrans et al. (2025), Zhou et al. (2025), Quan et al. (2025), Lafon et al. (2025), Naeem et al. (2024), Tsitokana et al. (2023), Esparza et al. (2023), Jootar et al. (2026), Sun et al. (2025a)
CD33-TREM2 axis	CD33 overactivation suppresses aggregate clearance, TREM2 deficits impair phagocytosis and M2 polarization	TREM2 mRNA-delivering lipid nanoparticles; siCD33-loaded carriers; gene-delivering nanoparticles	Co-delivers TREM2 mRNA and siCD33/CRISPR modules via ionizable lipid nanoparticles to synergistically restore phagocytically competent microglial states	Satoh et al. (2025), Abdulkhaliq et al. (2026a), Che et al. (2026), Lin et al. (2025a), Huang et al. (2026a), Liu et al. (2014), Bai et al. (2025), Saber et al. (2024), Penney et al. (2024), Roamcharern and Yubolphan (2026)

*AD* Alzheimer's disease, *Aβ* amyloid-beta, *ASSIST* Aβ-targeting, STING-inhibiting, and immunomodulatory nanomaterials, *cGAS* cyclic GMP-AMP synthase, *DAMPs* damage-associated molecular patterns, *IL* interleukin, *IRF3* interferon regulatory factor 3, *mtDNA* mitochondrial DNA, *NF-κB* nuclear factor kappa-light-chain-enhancer of activated B cells, *NIM* nano-immunomodulator, *NLRP3* NLR family pyrin domain containing 3, *PD* Parkinson's disease, *PINK1* PTEN-induced kinase 1, STAT1, signal transducer and activator of transcription 1, *STING* stimulator of interferon genes, *TNF-α* tumor necrosis factor-alpha, *TREM2* triggering receptor expressed on myeloid cells 2

Across NIM platforms, scalable manufacturing, cost-effectiveness, and standardized characterization remain major barriers to clinical adoption (Dipankar et al. (2025). Advanced systems such as nanobody conjugates and engineered exosomes require specialized production workflows that may restrict large-scale implementation (Lankoff et al. (2024). Therefore, successful clinical translation will require not only continued mechanistic development but also reproducible manufacturing, comprehensive long-term safety evaluation, and scalable production strategies (Gkogkos et al. (2023) (Table 6).

**Table 6:** Translational challenges and platform-specific limitations of current NIM platforms

NIM platform category	Platform characteristics & context	Specific translational challenges & limitations	References
Polydopamine nanoparticles & biomimetic nanozymes	Provide intrinsic catalytic and antioxidant activities	Synthesis is highly sensitive to reaction conditions (pH, oxygen availability, precursor concentration), which affects particle size, surface properties, and biological activity	Battaglini et al. (2025)
	Possess reactive surfaces	Propensity to promote protein corona formation in biological fluids, altering targeting efficiency, biodistribution, and ROS-scavenging performance	Sun et al. (2024), Mei et al. (2025)
Carbon- and metal-based nanozymes	Intended for repeated therapeutic administration	Long-term biodegradation and clearance remain insufficiently characterized, raising concerns regarding systemic accumulation	Toader et al. (2024), Zheng et al. (2022)
Nanobody-based platforms	Offer high targeting specificity and reduced Fc-mediated immune activation	May still induce anti-drug antibody responses following repeated exposure	Ackaert et al. (2021), Pauw et al. (2023), Xenaki et al. (2021)
	Characterized by a very small physical size	Small size promotes rapid renal clearance, necessitating half-life extension strategies that increase overall design complexity	Ackaert et al. (2021), Peng et al. (2020), Zeng et al. (2023)
Exosome-based carriers	Provide high physiological biocompatibility	Limited by donor-cell variability, inconsistent cargo loading, and a lack of standardized production and quality-control procedures	Koh et al. (2023), Palakurthi et al. (2024)
	Possess a highly complex biological composition	Complex composition further complicates regulatory classification and manufacturing standardization	Lankoff et al. (2024)
Nanotheranostic systems	Utilize multifunctional architectures combining imaging agents, targeting ligands, therapeutic cargos, and responsive components	High structural complexity increases manufacturing demands, production cost, and batch-to-batch variability	Shanahan et al. (2025), Crist et al. (2021)
	Rely on retained imaging materials (e.g., metal-based contrast agents)	Long-term safety and biocompatibility of the retained imaging components require further comprehensive evaluation	Li et al. (2026)
Precision pathway interceptors (e.g., ASSIST-like systems)	Represent highly advanced targeted architectures	Application remains largely confined to early preclinical validation stages	Xie et al. (2023), Joseph and Nance (2022), Stucchi et al. (2026)
	Utilize a multi-component design	Adds regulatory complexity; each functional element may require independent assessment of safety, stability, and pharmacological performance	Zhang et al. (2025d)
Advanced NIM systems (e.g., nanobody conjugates & engineered exosomes)	Possess high structural and functional complexity	Require specialized production workflows that may severely restrict large-scale implementation	Lankoff et al. (2024)
All NIM Platforms (General)	Aim for widespread clinical adoption	Scalable manufacturing, cost-effectiveness, and standardized characterization remain major overarching barriers	Dipankar et al. (2025)
All NIM platforms (translational prerequisites)	Possess significant mechanistic advantages	Successful translation strictly requires reproducible manufacturing, comprehensive long-term safety evaluation, scalable production strategies, and continued mechanistic development	Gkogkos et al. (2023)

*Fc* Fragment crystallizable, *NIM* Nano-immunomodulator, *ROS* Reactive Oxygen Species

## Novel approaches and models in nano-immunomodulators

### Artificial intelligence-driven nanomedicine

Artificial intelligence (AI) and machine learning (ML) are emerging as powerful tools for accelerating NIM design in AD and PD by linking nanoparticle properties with biological outcomes (Krsek et al. 2025; Hosni et al. 2025). Supervised ML models, including random forests and gradient-boosting algorithms, can integrate physicochemical descriptors such as core composition, size, aspect ratio, surface charge, ligand density, and protein corona characteristics with experimental parameters, including BBB permeability, endothelial viability, microglial uptake, and cytokine responses (Li et al. 2025b; Sela et al. 2025). These approaches identify nanoparticle features associated with improved BBB transport and reduced toxicity, enabling rational optimization of NIM formulations beyond empirical screening (Li et al. 2025b; Tomitaka et al. 2023; Su et al. 2025).

ML-based ligand prediction platforms further support the selection of targeting molecules by evaluating peptides, antibodies, and aptamers for affinity and specificity toward disease-relevant targets, including Aβ plaques, α-synuclein aggregates, and microglial receptors such as TREM2 and CX3CR1 (Bansal et al. 2025; Rock et al. 2022; Liu et al. 2026b).

In parallel, deep learning and network-based approaches can integrate single-cell transcriptomic and proteomic datasets to characterize immune-cell states, identify therapeutic targets, and guide nanomedicine design (Xu et al. 2025c; He et al. 2024a, 2024b).

AI-driven workflows have also been applied in preclinical AD and PD nanomedicine research to prioritize nanoparticle formulations with favorable BBB properties and identify targeting strategies for advanced platforms, including exosome-mimetic vesicles and gene-delivery lipid nanoparticles (Krsek et al. 2025; Tomitaka et al. 2023). Collectively, these approaches provide a data-driven framework for improving the precision, efficiency, and translational potential of NIM development (Krsek et al. 2025; Hosni et al. 2025; Zheng et al. 2026).

### Next-generation pre-clinical models

To overcome the interspecies limitations and predictive failures of traditional animal models, emerging platforms accurately replicate human neurobiology.

#### Brain-on-a-chip

Brain-on-a-chip systems are preclinical microfluidic in vitro models that reconstruct the human neurovascular unit (NVU) using human-derived cells. By simulating blood flow and incorporating disease markers, they enable high-throughput, real-time evaluation of nanoparticle BBB permeability. Integrated biosensors continuously monitor pH, oxygen, cytokine release, and nanoparticle distribution to provide granular safety and efficacy data (Lee et al. 2021; Helm et al. 2016; Nguyen et al. 2026) (Table 7).

**Table 7:** Structural design, functional capabilities, and applications of advanced In Vitro neurobiology models

Pre-Clinical Model	Design & Structural Features	Functional Applications & Capabilities	References
Brain-on-a-Chip	Microfluidic Architecture: Reconstructs the human NVU in vitro using human-derived cells	Physiological Simulation: Simulates blood flow and incorporates relevant disease markers	Lee et al. (2021)
	Integrated Biosensors: Features continuous, real-time monitoring systems for pH, oxygen levels, cytokine release, and nanoparticle distribution	High-Throughput Evaluation: Enables real-time assessment of nanoparticle BBB permeability; provides granular safety and efficacy data	Lee et al. (2021)
3D Bioprinted Brain Tissues	Matrix Composition: Embeds NVU cells within tunable, brain-mimetic 3D matrices, overcoming 2D culture constraints	Phenotypic Preservation: Maintains native glial phenotypes	Lee et al. (2021)
	Perfusable Channels: Utilizes engineered channels to facilitate structural testing and fluid dynamics	Crosstalk & Stress Testing: Recreates essential multicellular crosstalk (e.g., neuron-glia signaling via NF-κB, NLRP3, and cGAS-STING); evaluates BBB integrity under inflammatory stress	Wang et al. (2025d), Aiyegbusi et al. (2026)
	Pathological Triggers: Incorporates specific disease markers (e.g., Aβ or α-synuclein)	Metabolic & Spatial Evaluation: Captures realistic metabolic shifts; allows for the direct spatial evaluation of stimuli-responsive nanocarriers	Qiu et al. (2025), Baghirov (2025), Tang et al. (2025)

*2D* Two-dimensional, *3D* Three-dimensional, *Aβ* Amyloid beta, *BBB* Blood–brain barrier, *cGAS* Cyclic GMP-AMP synthase, *NF-κB* Nuclear factor kappa-light-chain-enhancer of activated B cells, *NLRP3* NLR family pyrin domain containing 3, *NVU* Neurovascular unit, *STING* Stimulator of interferon genes, *α-synuclein* Alpha-synuclein

In the context of neuroinflammatory nanomedicine, NVU chips have been applied to systematically compare BBB traversal and neurovascular toxicity across different NIM formulations, quantifying changes in transendothelial electrical resistance, tight-junction protein expression, and cytokine profiles in response to polymeric nanoparticles, exosome-mimetic vesicles, and lipid-based carriers before progression to in vivo AD/PD models (Ferrari et al. 2025; Mao et al. 2024) (Table 7).

#### 3D bioprinted brain tissues

To overcome the limitations of conventional 2D cultures, 3D bioprinting enables the integration of NVU components within tunable, brain-mimetic matrices that better preserve native cellular phenotypes (Lee et al. 2021). These models reproduce essential multicellular interactions, including neuron-glia signaling pathways involving NF-κB, NLRP3, and cGAS–STING, while incorporating perfusable channels to evaluate BBB function under inflammatory conditions (Wang et al. 2025d; Aiyegbusi et al. 2026). The introduction of disease-relevant stimuli, such as Aβ or α-synuclein, further enables modeling of pathological metabolic changes and spatial assessment of stimuli-responsive nanocarrier behavior (Qiu et al. 2025; Baghirov 2025; Tang et al. 2025) (Table 7).

In NIM development, 3D bioprinted brain tissues provide human-relevant platforms for evaluating nanoparticle effects on microglial activation states, neuronal viability, and BBB integrity under AD- and PD-like inflammatory environments. These systems support dose–response analysis and multidimensional safety evaluation of advanced NIM platforms, including stimuli-responsive and gene-delivery nanocarriers, within a physiologically relevant neurovascular context (Wang et al. 2025d; Aiyegbusi et al. 2026; Qiu et al. 2025; Baghirov 2025; Tang et al. 2025) (Table 7).

### Convergent technologies

Neuroinflammatory nanomedicine is shifting toward programmable systems designed to actively restore microglial phagocytosis and metabolism, rather than merely suppressing cytokines.

#### Nanobody-drug conjugates

Camelid-derived nanobodies efficiently target cryptic CNS epitopes (e.g., conformation-specific oligomers) without Fc-driven immunogenicity, making them promising candidates for BBB-directed delivery. However, nanobody-drug conjugates remain largely at the preclinical stage. Conjugating them to drug-loaded nanoparticles enables precise dual-target localization. For example, pairing an anti-aggregate nanobody with an NLRP3 inhibitor (e.g., MCC950) halts localized NF-κB signaling and oxidative stress at sites of maximal inflammasome priming (Sela et al. 2025; Rafiei et al. 2025). Advanced constructs utilize BBB shuttles and microenvironment-responsive linkers, though rapid renal clearance and durable CNS exposure remain key challenges (Feiten et al. 2026; Liao et al. 2025; Han et al. 2024).

#### Gene-delivering nanoparticles

Gene-delivery platforms, particularly lipid nanoparticles (LNPs), use engineered ionizable lipids and targeting ligands to enhance microglial uptake and promote endosomal escape in early preclinical systems (Bai et al. 2025; Saber et al. 2024; Penney et al. 2024). These approaches aim to modulate disease-relevant regulatory pathways, including the CD33–TREM2 axis. TREM2 mRNA delivery has provided proof-of-concept evidence for enhancing phagocytic microglial functions through TYROBP–SYK–PI3K–AKT signaling while reducing NLRP3- and cGAS–STING-associated inflammatory responses in experimental models (Roamcharern and Yubolphan 2026). Conversely, targeted siRNA or CRISPR-based strategies have been explored to suppress inhibitory CD33 signaling; however, their long-term safety, durability, and cell-type specificity remain unresolved (Roamcharern and Yubolphan 2026).

Co-delivery strategies combining TREM2 enhancement with CD33 inhibition represent an emerging approach to microglial reprogramming. However, efficient BBB delivery, endosomal escape, and determination of safe therapeutic windows for immune modulation require further validation in human iPSC-derived models and translational studies (Roamcharern and Yubolphan 2026). Table 5 summarizes the mechanistic relationships between molecular targets and corresponding nanotherapeutic strategies discussed in this review.

Integrating targeted nanocarriers, stimuli-responsive delivery systems, and nucleic acid-based microglial reprogramming provides a potential framework for next-generation precision neuroimmunotherapy. Table 8 summarizes the major NIM platforms according to nanoparticle type, targeting strategy, therapeutic mechanism, advantages, and limitations.

**Table 8:** Comparative classification of nano-immunomodulator platforms in AD and PD

Nanoparticle type	Targeting strategy	Therapeutic mechanism	Advantages	Limitations	References
Polydopamine nanoparticles	Intrinsic redox-active ROS scavenging; passive BBB interaction	Antioxidant and anti-inflammatory effects; suppression of pro-inflammatory signaling	Simple composition; broad mechanistic utility; intrinsic bioactivity	Batch variability; incomplete biodegradation profiling; limited clinical validation	Huang et al. (2026a), Ryu et al. (2018), Wang et al. (2026a), Duță et al. (2025), Duan et al. (2026), Ceccarelli et al. (2025), Wang et al. (2026b), Golchin et al. (2017)
Biomimetic nanozymes	Enzyme-mimetic catalytic activity; surface biomimicry	ROS neutralization; mitigation of mitochondrial stress and inflammasome priming	Multifunctional antioxidant capacity; may support mitophagy-related mechanisms	Composition-dependent reproducibility; uncertain long-term biodistribution; scale-up challenges	Chen et al. (2026), Godugu et al. (2025), Amniouel et al. (2025), Ma et al. (2025b), Xia et al. (2025), Zha et al. (2024)
Nanobody-based carriers	Ligand-mediated BBB traversal; disease-epitope targeting	Localized delivery of drugs or inhibitors to pathological CNS sites	High specificity; small size; reduced Fc-mediated effects	Rapid clearance; manufacturing complexity; limited in vivo durability	Lafon et al. (2025), Naeem et al. (2024), Tsitokana et al. (2023), Esparza et al. (2023), Jootar et al. (2026), Sun et al. (2025a)
Exosome-based nanocarriers	Natural vesicle tropism; surface engineering	Cargo delivery of miRNAs, siRNAs, small molecules, or proteins	High biocompatibility; native membrane properties; potential BBB crossing	Heterogeneity; low loading efficiency; standardization and regulatory constraints	Huang et al. (2026b), Onohuean et al. (2025), Évora et al. (2025), Feng et al. (2025), An et al. (2025), Shahlaei et al. (2025), Abdulkhaliq et al. (2026b), Kudpaje et al. (2025), Ke et al. (2026), Salarpour et al. (2025), Wang et al. (2024)
Nanotheranostics	Stimuli-responsive and imaging-guided targeting	Combined diagnosis and controlled release of therapeutic payloads	Real-time monitoring; localized delivery	Multi-component complexity; cost; uncertain long-term safety	Kochman et al. (2026), Zhao et al. (2025a), Sun et al. (2025b), Araujo et al. (2026), Prasanth et al. (2025), Ballance et al. (2019), Liu et al. (2026a), Zhang et al. (2023a), Zhang et al. (2023b), Guo et al. (2024b), Zhao et al. (2025b), Tian et al. (2025c)
Precision pathway interceptors	Disease-node targeting; multi-payload co-delivery	Simultaneous modulation of cGAS-STING, NLRP3, or A-related pathways	Mechanistically integrated design	Early preclinical status; regulatory complexity; limited validation	Xie et al. (2023), Chung et al. (2024), Hettiarachchi et al. (2019), Mitchell et al. (2021), Topal et al. (2020), Lin et al. (2025b), Ren et al. (2025), Younis et al. (2022)
Gene-delivering LNPs	Ligand-directed RNA delivery; endosomal escape optimization	Delivery of mRNA, siRNA, or CRISPR payloads to reprogram microglia	Modular; adaptable; strong mechanistic specificity	Delivery efficiency; off-target concerns; limited CNS clinical translation	Bai et al. (2025), Saber et al. (2024), Penney et al. (2024), Roamcharern and Yubolphan (2026)

*Aβ* Amyloid Beta, *BBB* Blood–Brain Barrier, *cGAS* Cyclic GMP-AMP Synthase, *CRISPR* Clustered Regularly Interspaced Short Palindromic Repeats, *Fc* Fragment crystallizable, *LNPs* Lipid Nanoparticles, *miRNA(s)* Micro Ribonucleic Acid(s), *NLRP3* NOD-, LRR- and Pyrin Domain-Containing Protein 3, *ROS* Reactive Oxygen Species, *siRNA(s)*: Small Interfering Ribonucleic Acid(s), *STING* Stimulator of Interferon Genes

## Research gaps and future prospects

Despite significant advances in NIM development for AD and PD, major translational and mechanistic challenges remain, requiring coordinated progress across materials science, neuroimmunology, artificial intelligence, and clinical research. Many current NIM platforms are limited by complex multicomponent architectures, restricted manufacturing scalability, and insufficient batch-to-batch reproducibility. Variations in core composition, surface functionalization, and protein corona formation can substantially affect BBB transport, biodistribution, and immune responses.

Many preclinical nanoplatforms also accumulate substantially in peripheral organs, particularly the liver and spleen, with prolonged retention in the reticuloendothelial system, raising concerns about long-term safety and potential disruption of physiological microglial functions. Additional challenges include heterogeneous particle size distributions and incomplete characterization of degradation products, which complicate regulatory assessment and prediction of chronic CNS effects.

Addressing these platform-level limitations will require standardized physicochemical characterization, scalable manufacturing approaches, and comprehensive long-term toxicological evaluation. Resolving these challenges alongside remaining mechanistic uncertainties will be essential for developing safe, reproducible, and clinically translatable NIM-based neuroimmunotherapies for AD and PD.

### Microglial heterogeneity and NIM selectivity

Current NIMs should move beyond the binary M1/M2 paradigm and instead account for a broad continuum of microglial states, including disease-associated microglia DAM, activated response microglia ARM, homeostatic microglia, and other transcriptionally defined subpopulations identified through single-cell transcriptomics. Shifting the M1-to-M2 balance risks suppressing neuroprotective phenotypes. Future NIMs must selectively engage pathological subtypes at single-cell resolution using specific surface markers and transcription factor profiles.

### Dual targeting of the cGAS-STING-NLRP3 node

Although cGAS-STING activation cross-primes the NLRP3 inflammasome, intensifying neurodegeneration, no clinically validated nanoplatform simultaneously inhibits both pathways in vivo. Future combinatorial interceptors must co-deliver STING inhibitors and NLRP3 antagonists (e.g., MCC950) at disease-specific stoichiometric ratios, potentially utilizing cGAMP-triggered release.

### Gut-brain neuroinflammatory axis

Current nanomedicines address the gut or CNS in isolation, limiting their capacity to modulate dysbiosis-driven systemic-to-central inflammatory relays. Future progress is most likely to come from combining gut-directed anti-inflammatory interventions, SCFA-supportive approaches, and BBB-crossing nanocarriers within a staged therapeutic framework. At present, microbiome-responsive nanomedicine should be regarded as a promising but still early-stage concept, requiring mechanistic validation in longitudinal models that track both microbial composition and CNS inflammatory readouts.

### Modeling the peripheral-CNS immune interface

Current preclinical tools (e.g., static brain-on-a-chip or 3D tissues) fail to replicate the dynamic, multi-stage extravasation of peripheral immune cells, hindering the evaluation of NIMs targeting chemokine-dependent infiltration (e.g., blocking CCR2 or CXCR1/2). To address this, platforms must integrate perfusable vascular channels populated with PBMCs alongside CNS constructs. Crucially, these systems need a dynamically responsive BBB that degrades progressively under inflammatory stress (cytokines, ROS, MMPs) to mirror human pathology. Furthermore, models must incorporate iPSC-derived cells from patients with distinct genetic variants (e.g., ApoE4, LRRK2) to capture genotype-stratified infiltration dynamics absent in animal models.

### Microglial metabolic reprogramming as a target

Activated microglia shift to glycolysis via HIF-1, driving ROS overproduction and NLRP3 activation while impairing mitophagy. NIMs that reprogram metabolism toward oxidative phosphorylation remain exploratory, although this axis is increasingly supported by preclinical evidence. Future work should prioritize metabolically informed nanoplatforms that can be tested in iPSC-derived systems and organoid-based models before translation to in vivo disease settings.

### CD33-TREM2 axis: clinical translation

Co-delivering CD33 silencers and TREM2 mRNA via a single nanoparticle remains untranslated. Major barriers include undefined stoichiometric balances to avoid maladaptive overactivation, low endosomal escape efficiency in microglia, and poor CNS-selective delivery across early-disease BBBs. Future research must define safe upregulation windows, utilize inflammation-responsive promoters, and validate constructs in iPSC-derived models capturing variants like TREM2 R47H.

### AI-driven NIM design: from prediction to validation

A gap exists between in silico AI predictions and in vivo validation, as training datasets often derive from simplified models lacking neurovascular complexity. In the near term, AI should be used mainly for formulation ranking, feature selection, and hypothesis generation rather than as a substitute for experimental validation. Federated or multimodal learning pipelines integrating 3D bioprinting, organoid pharmacokinetics, and human neuroimaging may improve predictive accuracy, but these approaches still require extensive benchmarking against robust preclinical data.

### Nanotheranostics: real-time monitoring

Existing nanotheranostics lack spatial resolution to detect individual plaques or Lewy bodies in vivo, and their responsive release mechanisms are not yet validated in human brain tissue. The most realistic next step is to refine multimodal imaging-enabled systems that combine established diagnostic probes with preclinical stimulus-responsive release, while avoiding assumptions of immediate clinical utility. Development should prioritize reproducibility, sensitivity to disease-associated microenvironments, and compatibility with longitudinal safety assessment.

### Long-term safety and regulatory translation

Most preclinical studies assess short-term outcomes, leaving the chronic consequences of CNS nanoparticle accumulation unknown. Future priorities include longitudinal biocompatibility studies in aged disease models and establishing standardized regulatory frameworks (aligned with FDA and EMA guidelines). Additionally, scalable synthesis protocols for complex constructs (nanobody conjugates, exosome carriers) are prerequisite for Phase I/II clinical trials.

#### Comparative perspective with existing therapeutics

The translational potential of NIMs can be better understood by comparison with current and investigational therapies for AD and PD (Lei et al. 2024b; Kim and Lee 2024). Conventional anti-inflammatory agents, including corticosteroids and nonsteroidal anti-inflammatory drugs, can reduce systemic inflammation but are limited by poor CNS selectivity, incomplete modulation of disease-relevant pathways, and systemic toxicity (Mallick et al. 2025; Coll et al. 2015). Similarly, small-molecule inhibitors of inflammatory signaling pathways often show promising in vitro activity but face limited BBB penetration, pharmacokinetic instability, and insufficient in vivo durability (Coll et al. 2015).

Direct NLRP3 and STING inhibitors offer promising strategies for targeting upstream inflammatory amplification pathways; however, their development is constrained by incomplete target validation, context-dependent pathway functions, and concerns about long-term immune modulation (Zheng et al. 2024; Gu et al. 2024).

Monoclonal antibodies provide high target specificity and have advanced neurodegenerative disease research, particularly for extracellular pathological targets (Lei et al. 2024b; Pardridge 2023). However, their large molecular size, high production costs, limited BBB penetration, and dependence on receptor-mediated transport restrict their ability to modulate intracellular inflammatory pathways (Pardridge 2023; Mahanta et al. 2025). In contrast, NIM platforms can integrate targeting ligands, protective cargo encapsulation, BBB-targeting strategies, and stimulus-responsive release within a single system (Kim and Lee 2024; Ralvenius et al. 2024). This flexibility enables potential co-delivery of anti-inflammatory agents, RNA therapeutics, or pathway inhibitors to specific CNS cell populations, including microglia and endothelial cells (Kim and Lee 2024; Mahmoudi et al. 2023). However, these advantages remain largely theoretical because most NIM platforms are still in preclinical development and face challenges in manufacturing reproducibility, batch consistency, protein corona formation, long-term CNS safety, and regulatory standardization (Gkogkos et al. 2023; Zhang et al. 2023c).

Conventional small molecules and selected monoclonal antibodies remain the most clinically mature approaches because of established manufacturing processes and regulatory frameworks (Gkogkos et al. 2023; Lei et al. 2024b). NIMs may provide greatest value where conventional strategies are insufficient, particularly for multi-target modulation, localized BBB-directed delivery, and stimulus-responsive regulation of interconnected inflammatory pathways such as cGAS–STING and NLRP3 (Ralvenius et al. 2024; Đorđević et al. 2022). Thus, nanomedicine is not intended to universally replace existing therapies but to overcome specific biological and pharmacological limitations of single-agent conventional approaches (Gkogkos et al. 2023; Ralvenius et al. 2024) (Table 9).

**Table 9:** Comparative overview of nano-immunomodulators and current therapeutic approaches in AD and PD

Therapeutic modality	Advantages & therapeutic potential	Limitations & challenges	Current clinical maturity & role	References
Conventional anti-inflammatory agents (e.g., corticosteroids, NSAIDs)	Capable of reducing systemic inflammation	Poor CNS selectivity, incomplete modulation of disease-relevant pathways, and systemic toxicity	Existing therapeutics; insufficient for complex neuroinflammation	Mallick et al. 2025; Coll et al. 2015)
Small-molecule inhibitors (inflammatory signaling)	Demonstrate promising in vitro activity against inflammatory signaling pathways	Limited BBB penetration, pharmacokinetic instability, and insufficient in vivo durability	Clinically mature approach due to established manufacturing processes and regulatory frameworks	Gkogkos et al. 2023; Lei et al. 2024b; Coll et al. 2015)
Direct NLRP3 and STING inhibitors	Offer targeted strategies for upstream inflammatory amplification pathways	Incomplete target validation, context-dependent pathway functions, and concerns regarding long-term immune modulation	Investigational therapeutics	Zheng et al. 2024; Gu et al. 2024)
Monoclonal antibodies	Provide high target specificity; have significantly advanced neurodegenerative disease research (particularly for extracellular targets)	Large molecular size, high production costs, limited BBB penetration, dependence on receptor-mediated transport, and restricted ability to modulate intracellular pathways	Clinically mature approach with established regulatory and manufacturing frameworks	Gkogkos et al. 2023; Lei et al. 2024b; Pardridge 2023; Mahanta et al. 2025)
NIM platforms (architectural & delivery features)	Integrate targeting ligands, protective cargo encapsulation, BBB-targeting, and stimulus-responsive release; enable co-delivery of varied cargos (anti-inflammatories, RNA therapeutics, pathway inhibitors) to specific CNS cells (microglia, endothelial cells)	Advantages remain largely theoretical; restricted by protein corona formation and uncertainties regarding long-term CNS safety	Investigational; currently largely confined to preclinical development	Kim and Lee 2024; Ralvenius et al. 2024; Mahmoudi et al. 2023; Zhang et al. 2023c)
NIM platforms (manufacturing & translation)	—	Face significant barriers in manufacturing reproducibility, batch consistency, and regulatory standardization	Require resolution of comprehensive production and regulatory barriers prior to clinical translation	Gkogkos et al. 2023; Zhang et al. 2023c)
NIM platforms (clinical niche)	Overcome biological and pharmacological limitations of single-agent therapies; ideal for multi-target modulation, localized BBB-directed delivery, and stimulus-responsive regulation of interconnected pathways (e.g., cGAS–STING and NLRP3)	Not intended to universally replace existing therapies	Positioned to provide value specifically where conventional strategies remain insufficient	Gkogkos et al. 2023; Ralvenius et al. 2024; Đorđević et al. 2022)

*AD* Alzheimer's Disease, *BBB* Blood–Brain Barrier, *cGAS* Cyclic GMP-AMP Synthase, *NIMs* Nano-immunomodulators, *NLRP3* NOD-, LRR- and Pyrin Domain-Containing Protein 3, *NSAIDs* Nonsteroidal Anti-Inflammatory Drugs, *PD* Parkinson's Disease, *STING* Stimulator of Interferon Genes

#### Clinical trial landscape, regulatory considerations, and barriers to translation

Despite mechanistic advances in NIM platforms, their clinical translation in AD and PD remains at an early stage (Gkogkos et al. 2023; Song et al. 2024). Most approaches discussed in this review should therefore be considered preclinical or proof-of-concept rather than established therapeutic modalities (Gkogkos et al. 2023; Zhang et al. 2025d). To date, no nanoparticle-based immunomodulatory therapy has reached late-stage clinical validation for neurodegenerative indications (Gkogkos et al. 2023; Sol et al. 2025).

However, several nanocarrier platforms have entered early clinical evaluation (Kochman et al. 2026; Zheng et al. 2022) In AD, the liposomal formulation APH-1105 (NCT03806478) is being evaluated for intranasal delivery in mild-to-moderate disease, while tau-targeting liposomal vaccines ACI-35.030 and JACI-35.054 (NCT04445831) have advanced to Phase 2 studies (Kochman et al. 2026; Andreadou et al. 2026). In PD, the catalytic gold nanocrystal formulation CNM Au8 completed a Phase 2 trial (NCT03815916, REPAIR PD), and the PEGylated glutathione liposome 2B3-101 (Talineuren, NCT04976127) is undergoing Phase 1 evaluation to enhance BBB delivery (Kochman et al. 2026).

Polymeric PLGA nanoparticles delivering anti-tau siRNA (NCT04294499) remain in early clinical development (Kochman et al. 2026; Zheng et al. 2022). Collectively, these studies demonstrate the feasibility of nanocarrier-based approaches but focus primarily on safety, pharmacokinetics, and preliminary efficacy rather than disease-modifying outcomes (Andreadou et al. 2026).

Exosome-based nanotherapies remain less advanced despite their potential as BBB-compatible carriers for RNA therapeutics (Huang et al. 2026b). Clinical studies of exosome platforms in neurodegenerative diseases are limited to early-phase safety and feasibility assessments, with no exosome-based NIM yet evaluated specifically for neuroinflammatory modulation in AD or PD (Gao et al. 2025b). This limited progression reflects persistent challenges in reproducibility, cargo loading, manufacturing scalability, and quality control (Gao et al. 2025b). Similarly, BBB-targeted nanobody and nanobody–drug conjugate systems remain predominantly preclinical despite their favorable targeting properties and smaller molecular size than conventional antibodies (Ma et al. 2025b; Sun et al. 2025a) (Table 10).

**Table 10:** Clinical development landscape, trial status, and translational barriers of nanomedicine platforms in AD and PD

Platform category / candidate	Formulation & strategy	Target indication	Clinical development stage & trial identifier	Primary focus, key findings, & translational barriers	References
APH-1105	Liposomal formulation	Mild-to-moderate AD	Early clinical evaluation (NCT03806478)	Evaluated for intranasal therapeutic delivery	Kochman et al. 2026; Andreadou et al. 2026)
ACI-35.030 & JACI-35.054	Tau-targeting liposomal vaccines	AD	Phase 2 (NCT04445831)	Advanced to phase 2 clinical trials to target pathological tau	Kochman et al. 2026; Andreadou et al. 2026)
CNM-Au8	Catalytic gold nanocrystal formulation	PD	Phase 2 completed (NCT03815916, REPAIR PD)	Completed phase 2 trial evaluation	Kochman et al. 2026)
2B3-101 (Talineuren)	PEGylated glutathione liposome	PD	Phase 1 (NCT04976127)	Undergoing Phase 1 evaluation specifically aimed at enhancing BBB delivery	Kochman et al. 2026)
Anti-tau siRNA nanoparticles	Polymeric PLGA nanoparticles carrying anti-tau siRNA	Neurodegenerative diseases	Early clinical development (NCT04294499)	In early clinical evaluation for targeted anti-tau RNA delivery	Kochman et al. 2026; Zheng et al. 2022)
Early clinical nanocarriers (general assessment)	Various nanocarrier platforms	AD and PD	Early clinical evaluation (phase 1 / phase 2)	Demonstrates clinical feasibility; trials primarily focus on safety, pharmacokinetics, and preliminary efficacy rather than disease-modifying outcomes	Kochman et al. 2026; Zheng et al. 2022; Andreadou et al. 2026)
Exosome-based nanotherapies	BBB-compatible exosome carriers for RNA therapeutics	Neurodegenerative diseases (AD and PD)	Early-phase safety & feasibility assessments	Less advanced; no exosome platform has been evaluated specifically for neuroinflammatory modulation in AD or PD. Progression is restricted by persistent challenges in reproducibility, cargo loading, manufacturing scalability, and quality control	Huang et al. 2026b; Gao et al. 2025bGao et al. 2025b)
BBB-targeted nanobody systems	Nanobody and nanobody–drug conjugate systems	Neurodegenerative diseases	Predominantly preclinical	Possess smaller molecular size than conventional antibodies and favorable targeting properties, but remain mostly in preclinical development	Ma et al. 2025b; Sun et al. 2025a)

*AD* Alzheimer's Disease, *BBB* Blood–Brain Barrier, *PD* Parkinson's Disease, *PEG* Polyethylene Glycol, *PLGA* Poly(lactic-co-glycolic acid), *siRNA* Small Interfering Ribonucleic Acid

Regulatory translation of NIM platforms is further complicated by the lack of dedicated approval pathways for CNS-directed nanomedicines. Regulatory agencies, including the FDA and EMA, currently evaluate these products within existing drug, biologic, or combination-product frameworks, which may not fully accommodate complex multifunctional nanocarriers. Exosome-based products present additional classification challenges because of their biological origin and variable composition, complicating chemistry, manufacturing, and controls (CMC) requirements. Increasing emphasis is placed on standardized characterization of nanoparticle properties, including size distribution, surface charge, protein corona composition, and longitudinal biodistribution, although many academic NIM platforms have yet to generate these datasets.

Four major barriers currently limit clinical translation. First, human-relevant BBB targeting efficiency remains insufficiently demonstrated because rodent models may not accurately predict human CNS delivery. Second, reproducible GMP-compatible manufacturing remains challenging, particularly for complex systems such as exosomes and nanobody conjugates. Third, limited long-term data on CNS biocompatibility, degradation, and clearance complicate chronic safety assessment. Fourth, the high production costs and prolonged development timelines of multifunctional theranostic and precision-interceptor platforms may limit investment compared with established small molecules and monoclonal antibodies. Addressing these challenges will require coordinated efforts across nanomaterial engineering, regulatory science, and clinical development to establish standardized evaluation frameworks and translational pathways for CNS-directed nano-immunotherapies.

#### Critical appraisal of translational success and failure

Although NIMs have generated substantial mechanistic evidence in AD and PD, clinical translation remains limited by unresolved challenges in manufacturing reproducibility, CNS exposure, biodistribution control, and long-term safety. Many preclinical studies demonstrate target engagement in simplified cellular or rodent models, but these findings may not translate to human BBB physiology, aging-associated inflammatory environments, or chronic treatment conditions. Moreover, the structural complexity of multifunctional nanocarriers increases risks of batch variability, protein corona effects, and regulatory uncertainty, limiting near-term clinical advancement.

Among current platforms, intrinsically active systems such as polydopamine nanoparticles and selected biomimetic nanozymes may have relatively greater translational potential because of their simpler design, broader antioxidant and anti-inflammatory mechanisms, and reduced dependence on complex biological cargos. In contrast, exosome-based carriers, nanobody–drug conjugates, and gene-delivery nanoparticles offer greater biological specificity but face substantial challenges in manufacturing standardization, purification, cargo loading, and stability. Nanotheranostic systems and precision pathway interceptors remain promising but are less mature because of their complex architectures, increased production requirements, and regulatory challenges.

Several molecular targets also require careful therapeutic positioning because of their context-dependent functions. NF-κB is a key inflammatory regulator but also supports essential cellular homeostatic processes, making broad inhibition potentially harmful. Similarly, cGAS–STING and NLRP3 contribute to innate immune defense and cellular stress responses; therefore, complete and prolonged suppression may impair physiological immune functions. The CD33–TREM2 axis remains incompletely defined, as the optimal level of TREM2 activation needed to enhance phagocytosis without inducing maladaptive microglial responses is unknown. Likewise, although PINK1/Parkin-mediated mitophagy is an attractive target, the relative benefits of direct pathway activation versus indirect mitochondrial protection require further clarification.

Approaches with limited success include nonspecific systemic anti-inflammatory therapies lacking CNS selectivity, single-target interventions that fail to address the interconnected nature of mitochondrial dysfunction, inflammasome activation, and microglial dysregulation, and nanoplatforms that produce transient molecular effects without sustained functional improvement. Several proof-of-concept nanoconstructs have also failed to advance because of insufficient delivery efficiency, inconsistent performance across disease models, or unresolved safety concerns related to long-term CNS retention and immune modulation.

**Fig. 7:**
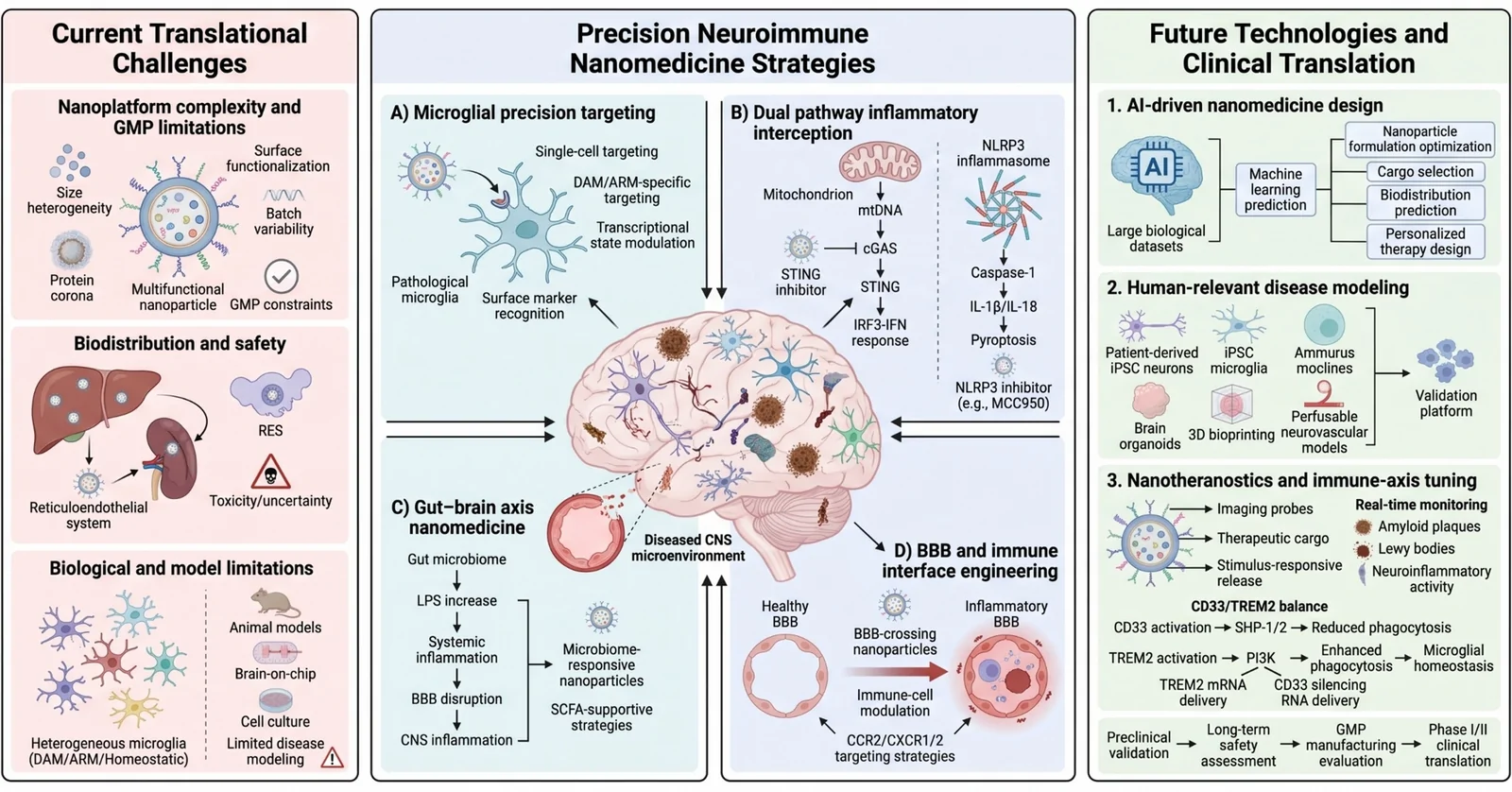
Future Perspectives of Neuroimmune Nanomedicine in Alzheimer’s and Parkinson’s Diseases: Overcoming Translational Barriers toward Precision CNS Immunotherapy. This schematic summarizes major challenges limiting the clinical translation of CNS-directed nanomedicines and highlights emerging approaches for precise, safe, and scalable neuroimmune regulation. Translational barriers include complex nanoplatform architectures, insufficient manufacturing reproducibility, variable physicochemical properties, protein corona formation, uncertain biodistribution, peripheral organ accumulation, and unknown long-term CNS safety. Additionally, conventional experimental models inadequately reproduce human neuroimmune complexity, such as microglial heterogeneity, dynamic BBB responses, and genotype-specific inflammatory phenotypes. Precision nanomedicine approaches aim to overcome these limitations by selectively targeting disease-associated microglial states beyond the simplified M1/M2 classification. Single-cell transcriptomic-guided strategies may enable subtype-specific modulation of pathological microglia while preserving homeostatic functions. The figure highlights multi-target inflammatory interception strategies, such as simultaneously modulating the cGAS–STING and NLRP3 inflammasome pathways using nanoplatforms that co-deliver pathway-specific inhibitors. These approaches aim to disrupt self-amplifying inflammatory circuits driven by mitochondrial dysfunction, mtDNA leakage, innate immune activation, and chronic neuroinflammation. Future NIM platforms may integrate gut-directed interventions with BBB-crossing nanocarriers to regulate microbiome-associated systemic inflammation and restore gut–brain immune communication. Furthermore, advanced neurovascular models incorporating perfusable vascular systems, patient-derived iPSCs, and dynamically responsive BBB platforms will improve predictions of immune-cell infiltration and therapeutic responses in human disease contexts. Emerging technologies including metabolically informed nanoplatforms, CD33–TREM2-targeted delivery systems, AI-guided nanoparticle design, and nanotheranostics may enable personalized microglial regulation, real-time disease monitoring, and therapeutic optimization. However, successful clinical translation requires integrating predictive modeling with experimental validation, standardized manufacturing, comprehensive toxicology, and updated regulatory frameworks for complex CNS nanomedicines. Abbreviations: AD: Alzheimer’s Disease, PD: Parkinson’s Disease, NIM: nano-immunomodulator; BBB: blood–brain barrier, cGAS: Cyclic GMP–AMP Synthase, STING: Stimulator of Interferon Genes, NLRP3: NOD-, LRR- and Pyrin Domain-Containing Protein 3, iPSC: Induced Pluripotent Stem Cell, AI: Artificial Intelligence, DAM: Disease-Associated Microglia, TREM2: Triggering Receptor Expressed on Myeloid Cells 2

Overall, successful translation will likely depend on balancing mechanistic sophistication with manufacturing feasibility, biological safety, and regulatory compatibility. Simpler, well-characterized, and functionally integrated systems may ultimately achieve greater clinical impact than highly complex multicomponent platforms unless advances in production, safety assessment, and regulatory frameworks progress in parallel. The major translational challenges limiting the clinical development of neuroimmune nanomedicine, together with emerging strategies for precision targeting, advanced disease modeling, AI-guided design, and clinical translation, are summarized in Fig. 7.

## Summary and conclusion

Integrating mechanistic understanding of chronic neuroinflammation including microglial state plasticity, NLRP3 activation, cGAS–STING signaling, impaired mitophagy, CD33–TREM2 dysregulation, peripheral immune infiltration, and gut–brain axis alterations with advanced NIM platforms provides a framework for developing multi-target and stimulus-responsive neuroimmunotherapies. However, therapeutic strategies will require disease-specific optimization because AD and PD differ in pathological substrates, anatomical vulnerability, and inflammatory organization. Current NIM approaches, including direct immunomodulatory nanomaterials, targeted nanocarriers, nanotheranostics, precision pathway interceptors, and gene-delivery lipid nanoparticles, demonstrate mechanistic feasibility primarily in preclinical models, while clinical translation remains limited.

Future progress will depend on addressing key challenges, including precise modulation of microglial subtypes beyond the M1/M2 classification, context-dependent regulation of pathways such as NLRP3 and cGAS–STING, effective gut–CNS targeting strategies, and definition of long-term CNS safety profiles. A more accurate therapeutic framework should regulate microglial functional states across the continuum of homeostatic, disease-associated, inflammatory, and repair-associated phenotypes rather than promote a simplified M1-to-M2 transition.

Ultimately, next-generation NIM-based therapies will require patient-stratified and genotype-informed approaches that incorporate disease-associated factors, including variants affecting pathways such as TREM2 and CD33, alongside individual biological characteristics such as microbiome composition. Human-relevant models, including iPSC-derived systems, brain-on-a-chip platforms, and 3D bioprinted tissues, may improve translational prediction. Nevertheless, clinical advancement will depend on prioritizing nanoplatforms with robust manufacturing processes, reproducible biological performance, favorable safety profiles, and durable in vivo efficacy. Addressing these requirements will be essential for translating nano-immunomodulators from mechanistic concepts into clinically viable precision neuroimmunotherapies.

## Data Availability

No datasets were generated or analysed during the current study.

## Abbreviations

Aβ: Amyloid-beta
AD: Alzheimer's disease
ApoE: Apolipoprotein E
BBB: Blood-brain barrier
CAT: Catalase
DAMPs: Damage-associated molecular patterns
GABA: Gamma-aminobutyric acid
HIF-1α: Hypoxia-inducible factor 1-alpha
IFN-β: Interferon-beta
IFN-γ: Interferon-gamma
IL-1β: Interleukin-1 beta
iPSC: Induced pluripotent stem cell
IRF3: Interferon regulatory factor 3
ITIM: Immunoreceptor tyrosine-based inhibition motif
LPS: Lipopolysaccharide
MMPs: Matrix metalloproteinases
mTOR: Mechanistic target of rapamycin
mtDNA: Mitochondrial DNA
NETs: Neutrophil extracellular traps
NIMs: Nano-immunomodulators
NIR-II: Near-infrared II
PD: Parkinson's disease
PD-1: Programmed cell death protein 1
PDNPs: Polydopamine nanoparticles
PET: Positron emission tomography
PI3K: Phosphoinositide 3-kinase
PINK1: PTEN-induced kinase 1
PPAR-γ: Peroxisome proliferator-activated receptor gamma
ROS: Reactive oxygen species
RVG: Rabies virus glycoprotein
SCFAs: Short-chain fatty acids
sdAb: Single-domain antibody
SHP-1/2: Src homology region 2 domain-containing phosphatases 1 and 2
SOD: Superoxide dismutase
SYK: Spleen tyrosine kinase
TGF-β: Transforming growth factor-beta
TLRs: Toll-like receptors
TNF-α: Tumor necrosis factor-alpha
TREM2: Triggering receptor expressed on myeloid cells 2
α-syn: Alpha-synuclein

## Declarations

### Declaration of AI use

The author affirms that the manuscript represents the author's original work and analysis. AI-based tools were used only to assist with language refinement and clarity. All scientific content, interpretations, figures, tables, and references were developed, verified, and written solely by the author. The author takes full responsibility for the accuracy, completeness, and integrity of the work.
